# A Thorough QT Study to Assess the Effects of Milvexian on Cardiac Repolarization in Healthy Participants

**DOI:** 10.1002/cpdd.70015

**Published:** 2026-01-19

**Authors:** Peter Zannikos, JongHanne Park, Anna Dari, Samiha Takhtoukh, Paul Levesque, Alexei N. Plotnikov, Amitava Mitra, Antoinette Ajavon‐Hartmann, Samira Merali, Juan Jose Perez Ruixo, Navin Goyal

**Affiliations:** ^1^ Johnson & Johnson Raritan NJ USA; ^2^ Johnson & Johnson Beerse Belgium; ^3^ Bristol Myers Squibb Princeton NJ USA; ^4^ Kura Oncology, Inc. Boston MA USA

**Keywords:** milvexian, pharmacokinetics, QT, repolarization, safety

## Abstract

Milvexian is an oral factor XIa inhibitor in development for prevention of major thromboembolic conditions. This randomized, double‐blind, placebo‐ and positive‐controlled, multiple‐dose, four‐period crossover study assessed the cardic safety of milvexian (including effects on the QT interval of the electrocardiogram), with a supporting in vitro component. Sixty‐six participants were enrolled. In each treatment period, participants received milvexian (100 or 200 mg) or placebo every 12 h for 4 days. A single‐dose moxifloxacin (400 mg) served as a positive control. Electrocardiographs and time‐matched pharmacokinetic samples were collected during each period. In mixed‐effects models, the upper limit of the two‐sided 90% confidence interval for the least squares means for change from baseline QTc (Fridericia [QTcF], as the primary correction method) for milvexian versus placebo (ΔΔQTc) was ˂10 ms at all time points after each milvexian regimen. In addition, there was no apparent relationship between ΔΔQTcF and plasma milvexian concentrations. Moxifloxacin response confirmed assay sensitivity. Milvexian inhibited human ether‐a‐go‐go‐related gene potassium, sodium, and L‐type calcium ion channel currents with weak‐to‐moderate potency at concentrations exceeding the highest mean unbound maximum plasma concentrations of TQT study participants. Milvexian regimens were safe and well tolerated. These data indicate that milvexian does not prolong the QTc interval at clinically relevant concentrations.

Vascular and thromboembolic diseases remain leading causes of death and disability worldwide.^1^, ^2^, ^3^ Anticoagulants are an important class of pharmacological agents that have demonstrated clinical benefits in the prevention and treatment of thrombosis and associated health disorders. These benefits are supported by a large body of clinical evidence, including randomized controlled trials and meta‐analyses.^4^, ^5^, ^6^, ^7^, ^8^ However, the role of anticoagulant therapy in the prevention and treatment of thromboembolism is continually limited by potential complications and risks associated with excess bleeding since administration of anticoagulants, such as vitamin K antagonists and direct‐acting oral anticoagulants, has been associated with increased bleeding risk.^9^, ^10^ Milvexian is a small molecule that inhibits the active form of clotting factor XI (FXIa) with high affinity and selectivity.^11^ Results from preclinical studies using animal models of arterial and venous thrombosis have shown that FXIa inhibition by milvexian has the potential to inhibit thrombin generation via the intrinsic pathway of coagulation while preserving hemostasis mediated by the extrinsic pathway.^12^, ^13^

The pharmacokinetic profile of milvexian has been characterized in healthy participants. Milvexian is rapidly absorbed after oral administration with a median time to maximum concentration (T_max_) of 2 to 4 h and a mean half‐life ranging from 11 to 18 h.^14^, ^15^ The bioavailability of milvexian was approximately 58% when orally administered as a spray‐dried dispersion formulation at a dose of 25 mg.^15^ Elimination of milvexian occurs through hepatic metabolism via cytochrome P450 (CYP) 3A4 and urinary excretion of unchanged drug. Milvexian also is a substrate for p‐glycoprotein.^16^ Lastly, the mean fraction of unbound milvexian in plasma of healthy participants was approximately 7.8% and 8.9%.^17^, ^18^

The efficacy and safety of milvexian were evaluated in two Phase 2 trials: AXIOMATIC‐TKR and AXIOMATIC‐SSP.^19^, ^20^ In the AXIOMATIC‐TKR trial, patients undergoing knee arthroplasty were randomized to receive one of the seven postoperative regimens of milvexian (25, 50, 100, or 200 mg twice daily; or 25, 50, or 200 mg once daily) or enoxaparin (40 mg once daily). The primary efficacy outcome was venous thromboembolism (VTE), which was a composite of asymptomatic deep vein thrombosis (DVT), confirmed symptomatic DVT or pulmonary embolism, or death from any cause. While milvexian dose dependently reduced the risk of VTE, no such relationship was observed for bleeding. In the AXIOMATIC‐SSP trial, patients who had acute ischemic stroke (IS) or high‐risk transient ischemic attack (TIA) were randomized in a 1:1:1:1:1:2 ratio to receive one of the five regimens of milvexian (25 mg once daily; or 25, 50, 100, or 200 mg twice daily) or placebo. Across all regimens of milvexian evaluated in the study, no significant dose‐dependent relationship was observed for the composite efficacy outcome of IS or covert brain infarction. In a pre‐specified analysis, milvexian was associated with fewer symptomatic IS compared with placebo at all doses except 200 mg twice daily.

Milvexian is being evaluated in the ongoing LIBREXIA Phase 3 program for stroke prevention after an acute IS or high‐risk TIA (LIBREXIA‐STROKE, NCT05702034), for reducing the risk of the composite of cardiovascular death, myocardial infarction, and IS in patients who have had a recent acute coronary syndrome (LIBREXIA‐ACS, NCT05754957), and for prevention of cardioembolic events in participants with atrial fibrillation (LIBREXIA‐AF NCT05757869). The milvexian dose regimen being evaluated in the LIBREXIA‐STROKE and ACS trials is 25 mg twice daily and is 100 mg twice daily for LIBREXIA‐AF.

Establishing cardiac safety of milvexian is of particular importance since the target population may be susceptible to the effects of any therapeutic agent affecting cardiac function. The QT interval (i.e., interval from the start of the Q wave to the end of the T wave) represents the duration of electrical depolarization and repolarization of the ventricular myocardium.^21^ Prolongation of the QT interval corrected for heart rate (QTc) is a recognized biomarker for potential proarrhythmic effects including fatal ventricular tachyarrhythmia, torsades de pointes, and is associated with increased risk for sudden cardiac death. Since drugs may influence ion channels and consequently lead to delay in cardiac repolarization, health authorities have mandated rigorous characterization of the effects of a new drug with systemic bioavailability on the QT/QTc interval.^22^, ^23^

Available data from Phase 1 studies of milvexian in healthy participants and participants with renal or hepatic impairment did not reveal any clinically important findings from electrocardiograms (ECGs).^16^, ^17^, ^18^, ^24^, ^25^ In these previously completed studies, up to 500 mg of milvexian was administered as a single dose or as a total daily dose for 14 consecutive days. However, the QT/QTc assessments conducted in these clinical studies were less extensive than those included in a conventional thorough QT (TQT) study and none of the studies included a positive control (i.e., moxifloxacin).

To support the overall cardiovascular safety assessment, the in vitro effects of milvexian on cardiac human ether‐a‐go‐go‐related gene (*hERG*) potassium, sodium, and L‐type calcium channel currents were tested. In addition, a TQT study was conducted to evaluate the potential for milvexian to prolong ventricular repolarization in healthy participants.

## Methods

### In Vitro Cardiovascular Safety Assessment

The effect of milvexian on cardiac ion channels that are involved in the depolarization/ repolarization processes was evaluated.^26^, ^27^ Milvexian was tested at 3, 10, and 30 µM (1879, 6265, and 18,794 ng/mL, respectively) in the hERG assay and at 10 µM in the sodium and L‐type calcium channel assays. Dimethyl sulfoxide (final concentration ≤0.3% in all assays) was used as a common vehicle. Milvexian was tested in three cells at each concentration in the ion channel assays. Membrane current recordings were made with a MultiClamp 700 series integrating patch‐clamp amplifier (Axon Instruments, Foster City, CA) using the whole‐cell variant of the patch‐clamp technique. Additional information of the methodology used to assess the effects of milvexian on potassium, sodium, and L‐type calcium currents is provided in the Supplemental Information.

### Thorough QT Study

The Phase 1 TQT study was conducted at a single clinical research unit in Merksem, Belgium (European Union Drug Regulating Authorities Clinical Trials 2021‐005030‐42).

The study protocol and informed consent were approved by an independent ethics committee (Comité voor Medische Ethiek UZA). The US Food and Drug Administration reviewed the protocol prior to the start of the study and deemed the design and analysis plan to be acceptable to characterize the effects of milvexian on the QTc interval. The study was conducted in accordance with the ethical principles that have their origin in the Declaration of Helsinki and are consistent with Good Clinical Practices and applicable regulatory requirements. Informed consent was obtained from all participants after they were told of the potential risks and benefits, as well as the investigational nature of the study.

### Study Design

The design of this study followed general design principles outlined in the International Council for Harmonisation (ICH) E14 guidance for the clinical evaluation of the QT/QTc interval with non‐antiarrhythmic drugs (Figure S1).^22^, ^23^ It was randomized, double‐blind, double‐dummy, placebo‐ and positive‐controlled, multiple‐dose, four‐intervention, four‐period, crossover and evaluated the influence of two milvexian regimens on QTc intervals in healthy participants, with moxifloxacin as a positive control. The study consisted of a screening phase, a double‐blind intervention phase with four intervention periods, and an end of study/early withdrawal assessment phase. A washout of ≥5 days separated the dosing between periods. An end of study/follow‐up visit was performed 7 to 10 days after discharge in the last intervention period. Eligible participants were admitted to the study site on Day −1 of each period and were discharged on Day 7 of each period after all study assessments were completed. Time‐matched extracts from Holter ECG recordings and pharmacokinetic samples were collected on Day 1 and Day 4 of each period.

### Participants

Eligible participants were males or females between 18 and 55 years of age inclusive, had a body mass index of 18 to 30 kg/m^2^ and a total body weight of >50 kg, and were non‐smokers. The participants were in general good health according to investigator opinion following a detailed medical history, physical examination, including vital signs measurements, 12‐lead ECG, and clinical laboratory tests at screening and Day −1 of the first intervention period. Women of childbearing potential were required to be using a highly effective method of contraception throughout the study and for at least 34 days after the last dose of study drug. Key exclusion criteria included disorders affecting cardiac function or repolarization (e.g., hypokalemia, history or family history of cardiac arrhythmias, or long QT syndrome) and defined ECG thresholds. Full inclusion and exclusion criteria, including restrictions on the use of concurrent medications that were unrelated to the study, are detailed in Table S1.

### Intervention Regimens

The milvexian regimen of 100 mg every 12 h (q12h) was included in the present study given that this is the highest dose regimen being investigated in the ongoing LIBREXIA Phase 3 program. The milvexian regimen of 200 mg q12h was intended to produce supratherapeutic concentrations in plasma and was given as an oral solution since results from a previous study in healthy participants indicated the oral bioavailability of milvexian is greater when administered as a solution relative to a solid dosage formulation.^15^ A single dose of moxifloxacin 400 mg was chosen as a positive control because it has an acceptable safety and tolerability profile in healthy participants and produces a well‐characterized increase in mean QTc.^28^, ^29^

The 100 mg dose of milvexian was administered as 100 mg oral capsules whereas the 200 mg dose was administered as 20 mL of a 10 mg/mL oral solution (80% w/w polyethylene glycol [PEG] 400 and 20% w/w polysorbate 80) filled in an amber glass vial. Matched placebo capsules and placebo solution that contained denatonium benzoate (Bitrex) were provided. Moxifloxacin was provided as commercially available 400 mg tablets that were over‐encapsulated.^30^ Moxifloxacin‐matched placebo was formulated as an over‐encapsulated tablet matching moxifloxacin. To maintain the double blind, participants received the same number of capsules and an oral solution, with matching placebos administered in place of active drug, across all periods.

On Day 1 of Period 1, before the first dose of study drug, eligible participants meeting study entry criteria were randomized to one of the four intervention sequence groups (ADBC, BACD, CBDA, or DCAB, per a William's design) and received one of the four interventions during each intervention period. In addition to milvexian and placebo, all participants received a single dose of 400 mg of moxifloxacin. A description of each intervention is provided below and in Figure S2.

Intervention A: Milvexian 100 mg capsule and milvexian solution‐matched placebo q12h on Days 1 to 4 plus moxifloxacin‐matched placebo on Day 4.

Intervention B: Milvexian 200 mg solution and milvexian capsule‐matched placebo q12h on Days 1 to 4 plus moxifloxacin‐matched placebo on Day 4.

Intervention C: Milvexian capsule‐matched placebo and milvexian solution‐matched placebo q12h on Days 1 to 4 plus moxifloxacin‐matched placebo on Day 4.

Intervention D: Milvexian capsule‐matched placebo and milvexian solution‐matched placebo q12h on Days 1 to 4 plus moxifloxacin 400 mg on Day 4.

Study drug was administered orally with 220 mL of water approximately q12h on Days 1 through 4, inclusive. On Day 1 and Day 4, participants fasted overnight prior to each morning administration. The fast continued for an additional 4 h after study drug administration. On Days 2 and 3, the morning dose of study drug was administered 30 min after a standard breakfast. A standard lunch, dinner, and light snack were provided at approximately 4, 8, and 9.5 h after the morning dose, respectively, on Days 1 to 4.

### Electrocardiogram Assessment

At least 1 h prior to the start of Holter ECG recording on Days 1 and 4 of each intervention period, participants were fitted with a 12‐lead Holter monitor. They rested for approximately 10 min before the scheduled time point for each ECG recording. On Day 1, triplicate recordings were obtained at 1 h, 45 min, and 30 min before administration of study drug (i.e., baseline) and at additional predefined time points up to 20 h after study drug administration. Triplicate recordings were also obtained on Day 4 at predefined time points up to 24 h after administration of study drug.

The 12‐lead Holter ECG was recorded using a digital 12‐lead dual‐lead ECG recorder (Surveyor Central System with S4 Mobile Monitors [Baxter‐Hillrom‐Welch Allyn]). Lead II was the primary lead with Lead V5 as the secondary lead if Lead II was not acceptable for measurement. A period of stable heart rate on the continuous Holter tracing within 4 min of the nominal ECG time point was identified. At each time point of ECG measurement, triplicate ECGs (three 10‐s ECG recordings at approximately 60‐s intervals) were extracted within 4 min.

Holter‐extracted ECGs were transferred electronically to an independent laboratory where they were assessed by cardiologists with extensive expertise in interpretation of ECGs using a high‐resolution, semi‐automatic (computer‐assisted) on‐screen caliper method. The Global Median Beat approach for measuring QT intervals was applied. This method uses an algorithm to derive representative beats from each of the 12 standard ECG leads. After medians were created for each lead, a global median was created by aligning the individual median beat. From there, the earliest onset to the latest offset is measured for all variables (PR [i.e., interval from the onset of PR wave to onset of the QRS complex, i.e., combination of Q, R, and S waves], QRS, and QT). Median beats were utilized to minimize the impact of noise present in any given single beat.

Cardiologists visually inspected the position of annotations placed by the automated algorithm within the ECG processing system. Wherever necessary, the annotations were manually adjusted to determine the interval duration. They were blinded to intervention group, intervention period, time point within the day, and the participant identifiers. A single cardiologist analyzed all ECGs from an assigned participant. Digital files for each 10‐s ECG with annotations of ECG intervals were stored in the e‐source system in the standard (HL7 XML) format.

The measured QT intervals were corrected for heart rate using Fridericia's as the primary correction method and additional, supportive correction methods (i.e., Bazett's, and population‐specific power [regression modeling of the baseline QT/interval between two ventricular depolarizations, RR, data pooled from all participants in the study]).^31^ Per participant, the average of the triplicate QTc intervals at each time point was used for the subsequent analyses. Baseline QTc for each participant was the average of the three sets of triplicate values obtained prior to dosing on Day 1 of each period. For each participant and regimen of milvexian, the change from baseline QT_C_ was calculated at each time point (ΔQTc). In addition, the difference in ΔQTc between milvexian and placebo (ΔΔQTc; i.e., ΔQTc for milvexian – ΔQTc for placebo) was calculated at each time point. Similarly, ΔΔQTc between moxifloxacin and placebo was calculated at each time point.

### Pharmacokinetic Assessment

The collection of pharmacokinetic samples was time matched with each corresponding ECG measurement. The interval between Holter ECG collection and pharmacokinetic blood sampling was ≤10 min. During the intervention periods, blood samples were collected from the participants on Day 1 (predose to 20 h postdose) and Day 4 (predose to 24 h postdose) in tubes that contained ethylenediaminetetraacetic acid.

The concentration of milvexian in plasma was measured by ICON Bioanalytical Laboratory (Assen, The Netherlands) using validated liquid chromatography–mass spectrometry/mass spectrometry methods. The validated range of this assay was 1.00 to 1000 ng/mL in plasma. This assay required 25 µL of plasma and used a stable isotope labeled (seven labels) internal standard. Sample preparation consisted of a protein precipitation with acetonitrile. Chromatographic separation was on a 2.1 × 50 mm 1.7 µm Waters Acquity UPLC C18 column with gradient elution by a mobile phase consisting of 0.1% acetic acid in water and acetonitrile at a flow rate of 1 mL/min. Detection was by tandem mass spectrometry (Sciex API 5500) operated in positive ionization. Quantification was based on multiple reaction monitoring of the transitions of m/z 626.1 → 280.1 for milvexian and 633.2 → 287.1 for the internal standard. The lower quantification limit of the bioanalytical assay was 1.00 ng/mL. Accuracy (i.e., percent bias) and precision (expressed as percent coefficient of variation), which were based on results of the analysis of four quality controls, were −0.7% to 5.0% and ≤4.5%, respectively.

Pharmacokinetic parameters of milvexian were calculated using noncompartmental methods with Phoenix WinNonlin version 8.3 (Certara L.P., Princeton, NJ).

### Safety and Tolerability

The safety and tolerability of milvexian were assessed by changes from baseline in physical examinations, vital sign measurements (heart rate, blood pressure, and body temperature), 12‐lead safety ECGs, and safety laboratory tests (i.e., hematology, coagulation tests, clinical chemistry, and urinalysis). Data that had the potential to unblind the intervention assignment, such as safety coagulation tests, were monitored by an independent, unblinded investigator to ensure participant safety while also preserving integrity of the blind. Baseline for laboratory evaluations, vital signs, and safety ECGs was defined as the last evaluation performed prior to the first administration of study intervention on Day 1 of Period 1. In addition, adverse events were monitored from the time the informed consent was signed until completion of the last study‐related procedure.

### Concentration‐QTc Modeling

A concentration‐QTc analysis was conducted using a pre‐specified linear mixed‐effects model. ΔΔQTcF and plasma milvexian concentration were the dependent and independent variables, respectively. Prior to analysis, all assumptions required for using the model were assessed: (1) no effect of milvexian on heart rate, (2) QTcF independence on heart rate, (3) lack of time delay between plasma milvexian concentration and ΔΔQTcF (i.e., hysteresis), and (4) linear relationship between ΔΔQTcF and plasma milvexian concentrations. The modeling was conducted using the pre‐specified model below.

(1) $$\begin{array}{ccc} & & \Delta \Delta \mathrm{QTcF}∼\left(\beta_{0}+\eta_{\mathrm{int}}\right)+\left(\beta_{1}+\eta_{\mathrm{slp}}\right)\\ & & \times \;\mathrm{Milvexian}\;\mathrm{concentration}+\beta_{2}\times \mathrm{QTcF}0+\varepsilon \end{array}$$

where β_0_ and β_1_ were the intercept and the slope of the linear concentration–effect relationship, respectively. Baseline QTcF at each study period (QTcF0) was included as a continuous covariate in the model, taken as the difference from population mean baseline (i.e., centered baseline), together with its associated fixed effect β_2_. The random effects η_int_ and η_slp_ quantified the inter‐individual variability on the intercept and the slope, respectively. The residual variability, ε, represented the random noise.

Based on the predefined model, the predicted ΔΔQTcF at the observed geometric mean maximum concentration at steady state (C_max,ss_) and its two‐sided 90% confidence interval (CI) were estimated by the following equations:

(2) $$\begin{array}{ccc} \mathrm{Estimated}\;\mathrm{mean}\Delta \Delta \mathrm{QTcF}(C_{\max,\mathrm{ss}}) & = & \beta_{0,\mathrm{Est}}+C_{\max,\mathrm{ss}}\\ & & \times \;\beta_{1,\mathrm{EST}} \end{array}$$

(3) $$\begin{array}{ccc} & & \mathrm{Estimated}\;\mathrm{standard}\;\mathrm{error}\;(\mathrm{SE})=\\ & & \;\sqrt{\mathrm{var}\left(\beta_{0,\mathrm{Est}}\right)+{C_{\max,\mathrm{ss}}}^{2}\times \mathrm{var}\left(\beta_{1,\mathrm{Est}}\right)+2\times C_{\max,\mathrm{ss}}\times \mathrm{cov}\left(\beta_{0,\mathrm{Est}},\beta_{1,\mathrm{Est}}\right)} \end{array}$$

(4) $$\begin{array}{ccc} 90\%\mathrm{CI} & = & \mathrm{Estimated}\;\mathrm{mean}\Delta \Delta \mathrm{QTcF}(C_{\max,\mathrm{ss}})\pm z\\ & & \times \;\mathrm{Estimated}\;\mathrm{SE} \end{array}$$

Where:

- β_0,Est_ and β_1,Est_ are the estimated intercept and slope, respectively;
- var(β_0,Est_) and var(β_1,Est_) are the estimated variance of intercept and slope, respectively;
- cov(β_0,Est_, β_1,Est_) is the estimated covariance between the intercept and slope;
- C_max,ss_ is the concentration of interest (predicted geometric mean C_max,ss_ at each dose level);
- z is the critical value of a standard normal distribution, for a confidence level of 90%.

The concentration‐QTcF analysis was based on a linear mixed‐effects model implemented in R (Version 3.6.2 or higher; Comprehensive R Network, http://cran.r‐project.org/ [2008]). The analyses were performed in a validated environment (internal High Performance Pharmacometrics Platform [HP3]), based on Good Automated Manufacturing Practice and in accordance with 21 CFR Part 11 and good clinical practice regulations.

### Statistical Analysis

The primary objective of the statistical analysis was to evaluate whether milvexian is non‐inferior to placebo with respect to its effect on QTc prolongation. A non‐inferiority criterion of 10 ms was selected for this evaluation.^22^, ^23^ Thus, the null hypothesis tested whether the largest difference in mean ΔQTc between milvexian and placebo (i.e., ΔΔQTc) was ≥10 ms against the alternative hypothesis that the largest difference in mean ΔQTc was <10 ms. Following the intersection–union principle, corresponding hypotheses were tested at each time point of measurement, at a one‐sided, 5% significance level. For each dose of milvexian, the effect of milvexian on QTc was considered as being non‐inferior to that of placebo if the upper limit of the two‐sided 90% CI (equivalent to one‐sided 95% CI) for the difference in mean ΔQTc between milvexian and placebo was <10 ms at all time points of measurement. Assay sensitivity was established if the lower limit of the two‐sided 97.5% CIs for the difference in mean ΔQTc between moxifloxacin and placebo (i.e., ΔΔQTc) exceeded 5 ms at ≥1 of the preselected time points (2, 3, 4, and 6 h postdose on Day 4).

The ΔQTc data obtained after administration of placebo, milvexian, and moxifloxacin were used in the statistical modeling. The average of the triplicate ECG measurements taken at each time point was used for the analysis. Mixed‐effect models were fitted to the ΔQTc interval as the dependent variable, and sequence, intervention, period, time point of measurement, and intervention by time point of measurement interaction as fixed effects and participant as a random effect. Using the least squares means and estimated intra‐participant variance, two‐sided 90% CIs were calculated for the difference in mean ΔQTc (i.e., ΔΔQTc) at each scheduled time point after administration of study intervention between (1) milvexian 100 mg q12h and placebo, (2) milvexian 200 mg q12h and placebo, and (3) moxifloxacin and placebo.

Sixty‐six participants were enrolled to ensure that at least 42 participants completed all required assessments. Based on previously conducted QT/QTc studies, the intra‐participant standard deviation (SD) for ΔQTc based on the primary correction method was assumed to be <10 ms. Using an SD of ≤9 ms, for a sample size of 42 participants, the probability that the upper limit of the two‐sided 90% CI for the difference in mean ΔQTc between each dose of milvexian and placebo (i.e., ΔΔQTc) at each time point of measurement would fall below 10 ms was estimated to be 80%, when the true difference in means equals 5 ms.

The incidence (count and percentage) of participants with a change from baseline in QTc >30 and >60 ms was tabulated for each treatment. The incidence of participants with any postdose QTc values >450 and >480 ms was also tabulated.

## Results

### In Vitro Cardiovascular Safety Assessment

To support the cardiovascular safety assessment, milvexian was assessed for potential in vitro effects on cardiac hERG potassium, sodium, and L‐type calcium channel currents using the whole‐cell variant of the patch‐clamp technique. Milvexian inhibited hERG/rapidly activating delayed rectifier K^+^ (IKr) currents by a mean (± SE of the mean [SEM]; n  =  3 cells) of 37.4% (3.0%), 57.3% (7.2%), and 78.1% (6.2%) at 3 µM (1879 ng/mL), 10 µM (6265 ng/mL), and 30 µM (18,794 ng/mL), respectively (Table 1). The cardiac hERG/IKr potassium channel half maximal inhibitory concentration (IC_50_) was 6.7 µM (4197 ng/mL). Milvexian (10 µM) at 1 Hz and 4 Hz stimulation frequencies inhibited cardiac sodium currents by a mean (± SEM; n = 3 cells) of 22.9% (4.4%) and 25.3% (4.6%), respectively. Milvexian (10 µM) inhibited cardiac calcium currents by mean (± SEM; n = 3 cells) of 13.9% (2.5%).

**Table 1:** Inhibitory Effect of Milvexian on hERG/IKr, Sodium, and Calcium Channel Currents

	hERG/K^+^ (IKr) (n = 3 cells)	Sodium (n = 3 cells)	L‐Type Calcium (n = 3 cells)
Milvexian, 3 µm (1879 ng/mL)	37.4 (3.0)	–	–
Milvexian, 10 µm (6265 ng/mL)	57.3 (7.2)	1 Hz: 22.9 (4.4)	13.9 (2.5)
		4 Hz: 25.3 (4.6)	
Milvexian, 30 µm (18,794 ng/mL)	78.1 (6.2)	–	–

hERG, human ether‐a‐go‐go‐related gene; IKr, delayed rectifier potassium current; SE, standard error of the mean.

Values are arithmetic mean % inhibition (SEM).

### Thorough QT Study

#### Participant Characteristics

Sixty‐six participants were enrolled in the study and randomly assigned to 1 of 4 possible intervention sequences (Table S2). Thirty‐five (53%) and 31 (47%) participants were female and male, respectively. Most participants were White (57 [86.4%]); the mean (SD) age, height, weight, and body mass index was 35.5 (11.0) years, 170.7 (9.42) cm, 73.6 (11.1) kg, and 25.2 (2.77) kg/m^2^, respectively.

Forty‐five participants completed all four periods as planned. Twelve participants discontinued prematurely: 8 of the 12 participants discontinued from the study because of a COVID‐19 infection and the remaining four  participants discontinued the study due to adverse events that were not COVID‐19 infection (moderate rash [n = 1], moderate contact dermatitis [n = 1], moderate psychological trauma [n = 1], and pregnancy [n = 1]). Five participants withdrew consent during the study, and four participants were withdrawn prematurely by the investigator (three participants due to noncompliance and one participant for disrespectful behavior).

#### Primary and Secondary Analyses

**Figure 1:**
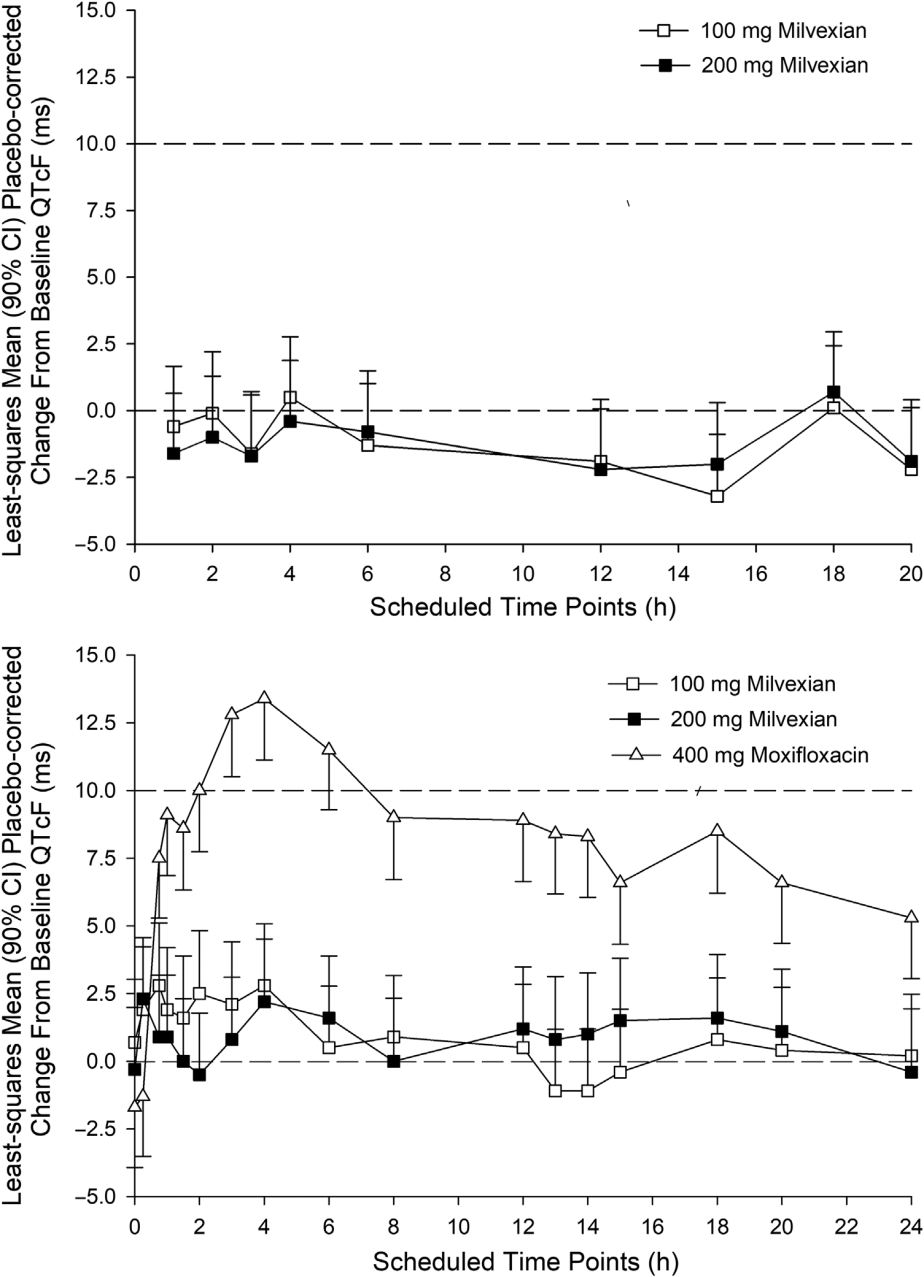
(A) Least squares mean (unidirectional 90% confidence interval) placebo‐corrected change from baseline QTcF for 100 and 200 mg of milvexian administered every 12 h: Day 1 (B) Least squares mean (unidirectional 90% confidence interval) placebo‐corrected change from baseline QTcF for 100 and 200 mg of milvexian administered every 12 h or a single dose of 400 mg of moxifloxacin: Day 4.

Compared with placebo, milvexian did not affect the QTcF at any postdose time points (Table 2 and Figure 1). The upper limit of the two‐sided 90% CI of the ΔΔQTcF was <10 ms. The highest upper limits were 5.16 and 4.57 ms for the 100 and 200 mg doses, respectively. Pre‐specified analyses showed consistent results using Bazett's and study‐specific correction methods (i.e., the upper limits of the 90% CIs for ΔΔQTc based on both methods for the milvexian regimens of 100 mg q12h and 200 mg q12h were <10 ms at all time points on Day 1 and Day 4; data not shown).

**Table 2:** Difference in ΔQTcF Between Milvexian (100 and 200 mg) or Moxifloxacin and Placebo at Each Time Point

Time Point	Milvexian 100 mg versus Placebo LS Mean (90% CI)	Milvexian 200 mg versus Placebo LS Mean (90% CI)	Moxifloxacin versus Placebo LS Mean (90% CI)
Day 1, 1 h	−0.6 (−2.86, 1.69)	−1.6 (−3.91, 0.64)	–
Day 1, 2 h	−0.1 (−2.41, 2.15)	−1.0 (−3.26, 1.29)	–
Day 1, 3 h	−1.6 (−3.91, 0.64)	−1.7 (−3.98, 0.58)	–
Day 1, 4 h	0.5 (−1.77, 2.79)	−0.4 (−2.67, 1.88)	–
Day 1, 6 h	−1.3 (−3.61, 0.94)	−0.8 (−3.06, 1.49)	–
Day 1, 12 h	−1.9 (−4.22, 0.35)	−2.2 (−4.52, 0.06)	–
Day 1, 15 h	−3.2 (−5.51, −0.94)	−2.0 (−4.25, 0.30)	–
Day 1, 18 h	0.1 (−2.23, 2.36)	0.7 (−1.62, 2.95)	–
Day 1, 20 h	−2.2 (−4.51, 0.06)	−1.9 (−4.15, 0.41)	–
Day 4, Predose	0.7 (−1.63, 2.99)	−0.3 (−2.58, 1.99)	−1.7 (−3.92, 0.61)
Day 4, 15 min	1.9 (−0.43, 4.19)	2.3 (−0.02, 4.57)	−1.3 (−3.51, 1.00)
Day 4, 45 min	2.8 (0.49, 5.11)	0.9 (−1.41, 3.18)	7.5 (5.29, 9.80)
Day 4, 1 h	1.9 (−0.39, 4.22)	0.9 (−1.41, 3.18)	9.1 (6.86, 11.37)
Day 4, 1 h 30 min	1.6 (−0.69, 3.92)	0.0 (−2.28, 2.31)	8.6 (6.33, 10.84)
Day 4, 2 h	2.5 (0.18, 4.80)	−0.5 (−2.82, 1.78)	10.0 (7.74, 12.25)
Day 4, 3 h	2.1 (−0.21, 4.40)	0.8 (−1.49, 3.10)	12.8 (10.51, 15.02)
Day 4, 4 h	2.8 (0.52, 5.16)	2.2 (−0.10, 4.52)	13.4 (11.13, 15.66)
Day 4, 6 h	0.5 (−1.77, 2.85)	1.6 (−0.70, 3.89)	11.5 (9.29, 13.80)
Day 4, 8 h	0.9 (−1.37, 3.25)	0.0 (−2.26, 2.33)	9.0 (6.71, 11.22)
Day 4, 12 h	0.5 (−1.84, 2.77)	1.2 (−1.11, 3.48)	8.9 (6.64, 11.15)
Day 4, 13 h	−1.1 (−3.38, 1.23)	0.8 (−1.46, 3.13)	8.4 (6.18, 10.69)
Day 4, 14 h	−1.1 (−3.39, 1.23)	1.0 (−1.33, 3.26)	8.3 (6.06, 10.57)
Day 4, 15 h	−0.4 (−2.73, 1.88)	1.5 (−0.79, 3.80)	6.6 (4.32, 8.83)
Day 4, 18 h	0.8 (−1.48, 3.13)	1.6 (−0.65, 3.94)	8.5 (6.22, 10.73)
Day 4, 20 h	0.4 (−1.93, 2.68)	1.1 (−1.19, 3.40)	6.6 (4.36, 8.87)
Day 5	0.2 (−2.08, 2.53)	−0.4 (−2.65, 1.94)	5.3 (3.05, 7.57)

CI, confidence interval; h, hour; LS, least squares.

The lower limit of the two‐sided 97.5% CIs (adjusted for multiplicity) for the adjusted mean difference in QTcF intervals between moxifloxacin 400 mg and placebo on Day 4 at 2, 3, 4, and 6 h ranged from 6.92 to 10.31 ms (data not shown), exceeding the lower protocol‐specified limit of 5 ms. Thus, QTc assay sensitivity was demonstrated. This was confirmed when using Bazett's and the population‐specific power QT correction methods (data not shown).

**Figure 2:**
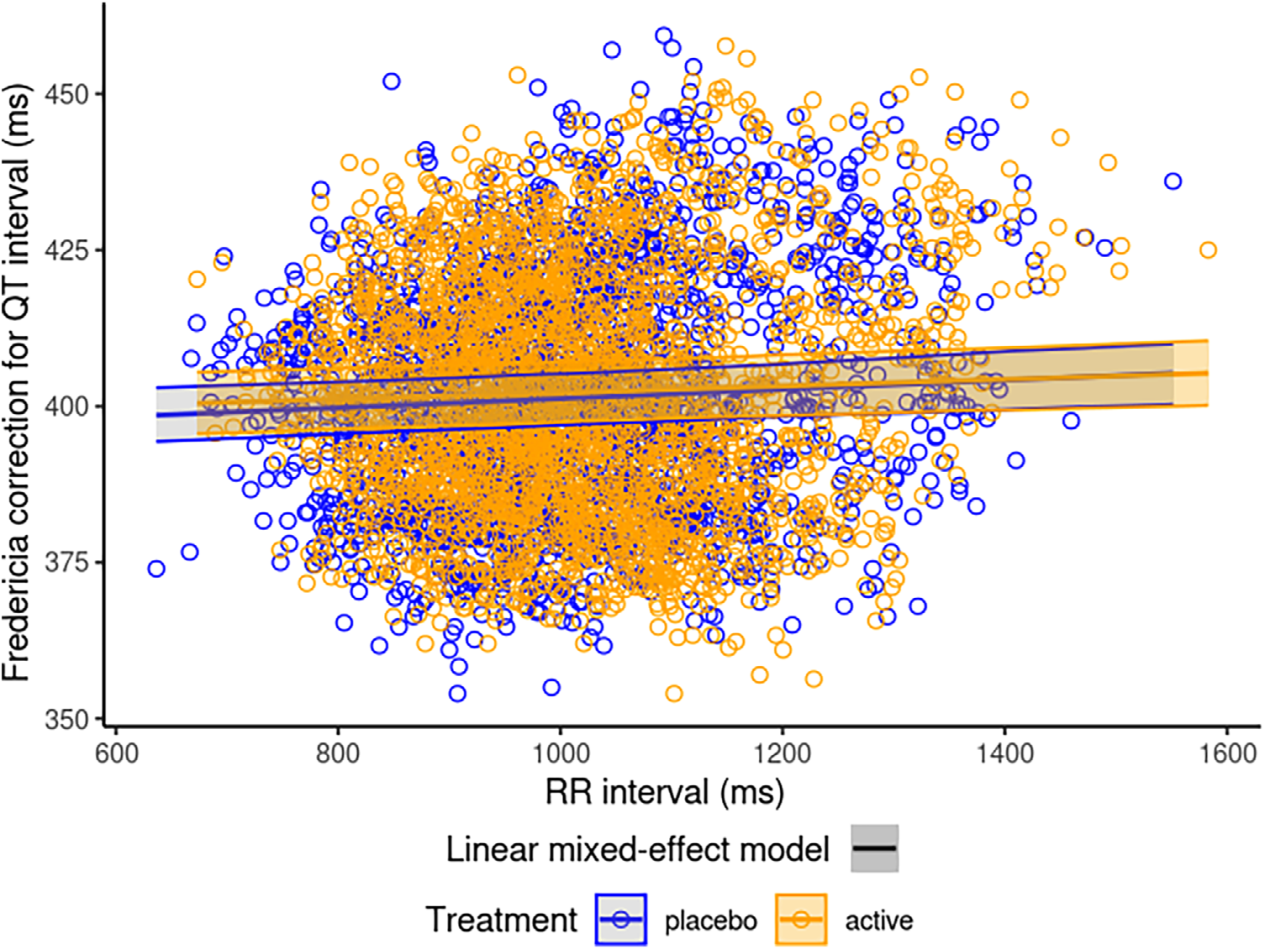
Scatterplot of QTcF versus RR. The slope of the linear regression was statistically significant (slope = 0.0073; 95% confidence interval: 0.0044, 0.010; *P* value = 7.35 × 10^−7^) but deemed too small to impact the concentration‐QTcF analysis. The solid lines represent the corresponding linear mixed model for the active (orange) and placebo (blue) treatments, respectively, with the 95% confidence interval. Open circles represent the active (orange) and placebo (blue) observations.

Administration of milvexian q12h did not alter heart rate. On Days 1 and 4, the time‐matched baseline‐adjusted least squares mean differences (90% CI) in heart rate between milvexian 100 mg q12h and placebo ranged from −1.9 beats per minute (bpm; −3.6, −0.10 bpm) to 0.3 bpm (−1.1, 1.7 bpm). The differences between milvexian 200 mg q12h and placebo ranged from −2.6 bpm (−4.2, −1.1 bpm) to 0.4 bpm (−1.2, 1.9 bpm). The scatterplot of QTcF versus RR using all individual baseline data (mean of triplicate values at 1 h, 45 min, and 30 min prior to each dosing on Day 1) from each period indicated that the relationship between QT and RR was effectively corrected using Fridericia's method (Figure 2).

No treatment‐emergent actual values of QTc >480 ms were observed during the study. Treatment‐emergent actual values of QTcF >450 to ≤480 ms were observed in 1 (1.8%) participant following 100 mg milvexian, 3 (5.5%) participants following 200 mg milvexian, 5 (8.5%) participants following moxifloxacin, and 5 (8.8%) participants following placebo. No treatment‐emergent changes from baseline of QTcF >60 ms were observed during the study. A treatment‐emergent change from baseline of QTcF >30 to ≤60 ms was observed in 1 (1.8%) participant following 100 mg milvexian, 2 (3.6%) participants following 200 mg milvexian, 9 (15.3%) participants following moxifloxacin, and 3 (5.3%) participants following placebo.

Treatment‐emergent QTc values and changes from baseline in QTc based on the Bazett's and population‐specific power QT correction methods were consistent with those summarized above (data not shown).

No consistent or clinically relevant changes over time were observed in heart rate, RR interval, PR interval, or QRS width. In addition, no T wave, U wave, or other morphologic findings were reported.

#### Pharmacokinetic Analyses

Plasma concentrations of milvexian could be measured from the first time point postdose and remained quantifiable until the end of the sampling interval. On Days 1 and 4, milvexian concentrations were 3 to 4 times higher for the 200 mg q12h regimen of milvexian solution, compared with the 100 mg q12h regimen administered as a capsule. For both milvexian regimens, concentrations in plasma were 2 to 3 times higher on Day 4, compared to Day 1. Mean (± SD) plasma concentration versus time profiles after oral administration of milvexian are shown in Figure S3. Key pharmacokinetic parameters of milvexian are presented in Table 3.

**Table 3:** Noncompartmental Pharmacokinetic Parameters of Milvexian in Plasma After Oral Administration of 100 mg (Capsule) or 200 mg (Solution) of Milvexian as a Single Dose and Every 12 h for 4 Days

Study Day	Milvexian 100 mg q12h for 4 Days (Intervention A)	Milvexian 200 mg q12h for 4 Days (Intervention B)
Day 1		
N	55	55
T_max_ (h)	4.00 (2.00 to 6.00)	1.98 (0.98 to 3.98)
C_max_ (ng/mL)	586 (201)	2310 (471)
AUC_12 h_ (ng h/mL)	3940 (1298)	14,506 (2164)
Day 4		
N	52	54
T_max_ (h)	3.49 (1.50 to 8.00)	1.48 (0.73 to 2.98)
C_max_ (ng/mL)	1444 (349)	4444 (798)
AUC_12 h_ (ng h/mL)	12,227 (3235)	36,387 (6747)

AUC_l2 h_, under plasma concentration time curve from time 0 to 12 h postdose; C_max_, maximum concentration; n, number of participants; T_max_, time to maximum concentration.

T_max_ is presented as median (minimum to maximum); C_max_ and AUC_12h_ are presented as arithmetic mean (SD).

#### Concentration‐QTc Modeling

**Figure 3:**
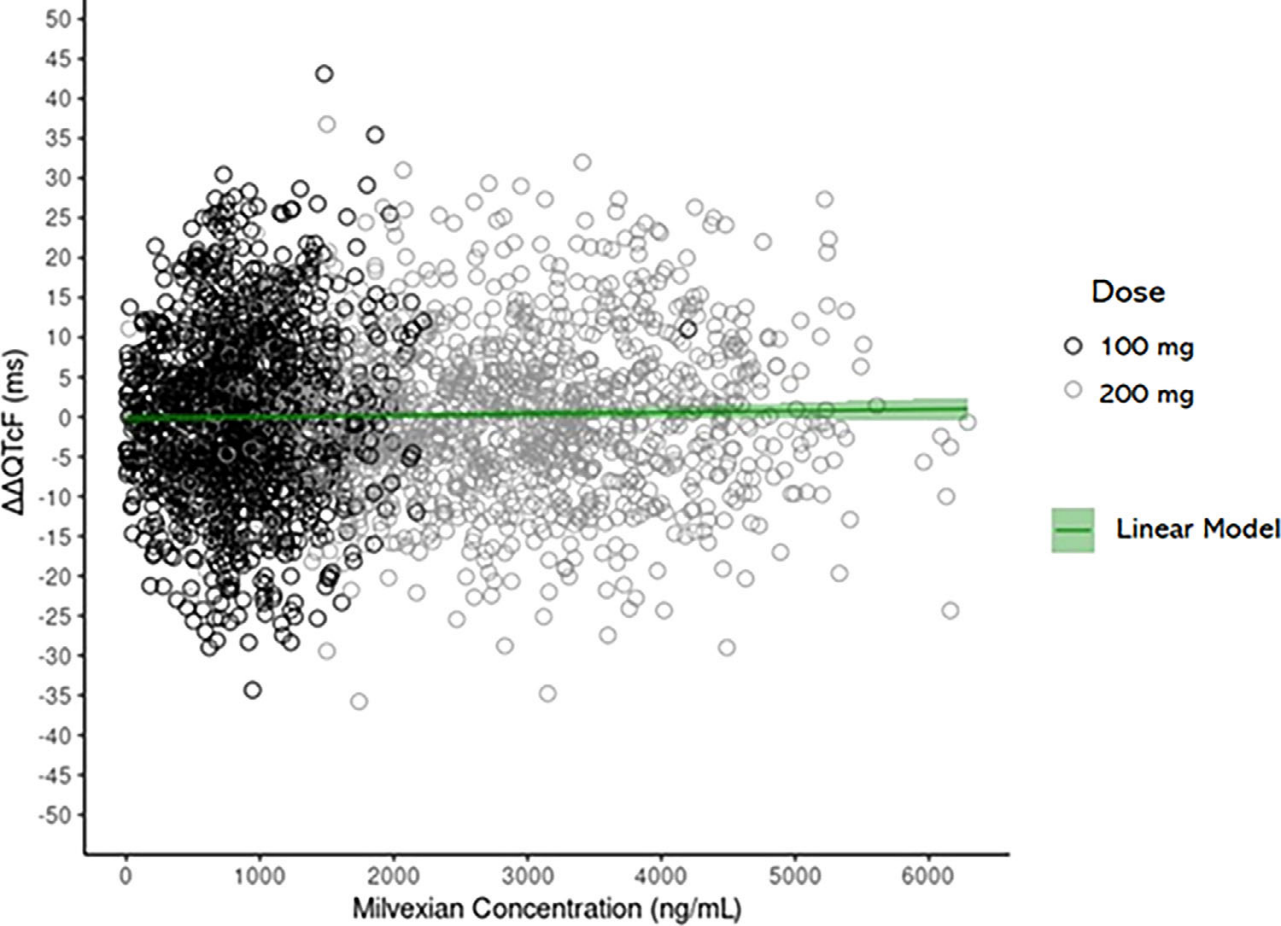
Scatterplot of observed ΔΔQTcF versus plasma milvexian concentrations.

The analysis dataset for concentration‐QTc modeling included 4630 time‐matched plasma milvexian concentration and ECG data points from the present TQT study. Prior to starting the concentration‐QTc modeling, all assumptions required to use the pre‐specified linear mixed‐effects model were tested: (1) milvexian did not have a clinically relevant effect on heart rate as the placebo‐corrected mean change from baseline heart rate was substantially <10 bpm (see section on primary and secondary analyses); similar results apply to the change from baseline heart rate (Figure S4), (2) scatter plots of QTc interval versus RR confirmed the appropriateness of the Fridericia correction (Figure 2), (3) hysteresis was not apparent as the mean (90% CI) of the observed ΔΔQTcF and milvexian plasma concentrations by sampling time point stratified by dose on Day 1 or Day 4 did not suggest a delayed effect (Figures S5 and S6), and (4) ΔΔQTcF and plasma milvexian concentrations could be described with a linear relationship (Figure 3). To quantitatively support this assumption, scatter plots of ΔΔQTcF and plasma milvexian concentration were fit with a linear and a quadratic model.^32^ The estimated quadratic term, when added to a linear mixed‐effect model, was not significant (*P* value of quadratic term =  0.929) at the two‐sided 5% alpha level and the models were compared via the Akaike's Information Criterion. Thus, the appropriateness of using a linear model was established.

Since the assumptions described above were met, the pre‐specified direct linear mixed‐effect model (Equation 1) was fitted to the data with ΔΔQTcF as the dependent variable. The fixed effect parameters included in the model were: the intercept, the slope associated with milvexian plasma concentrations, and influence of the baseline QTcF0 on the intercept. A random intercept and a random slope were included to account for between‐subject variability. Correlation between the two random effects was evaluated and the model without correlation was selected as it had a lower Akaike's Information Criterion.

**Figure 4:**
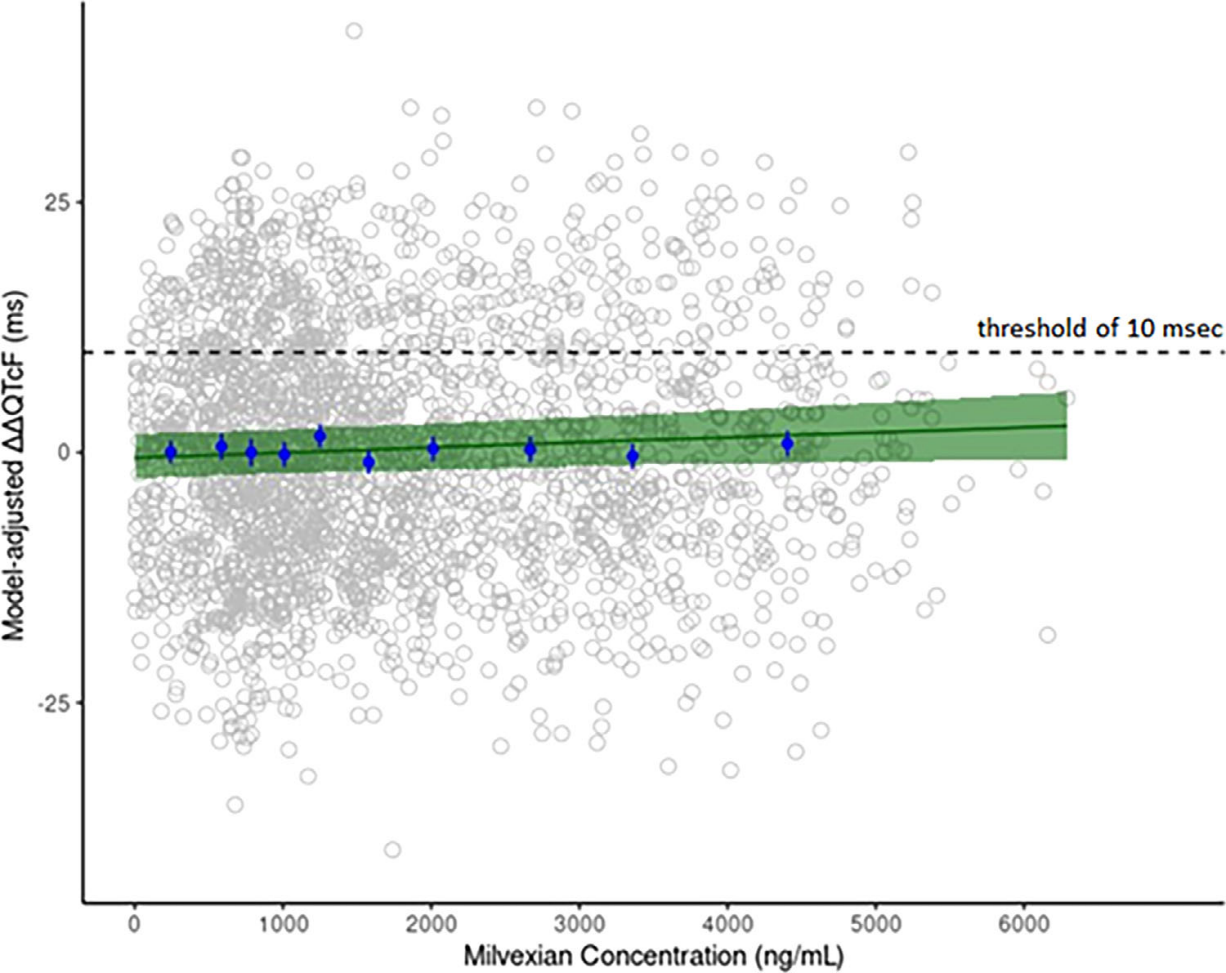
Model‐predicted ΔΔQTcF and observed model‐adjusted ΔΔQTcF versus plasma milvexian concentrations Solid line and shaded area represent the model‐predicted mean ΔΔQTcF and 90% confidence interval, respectively. Open circles are model‐adjusted observations by the non‐treatment‐related parameter (QTcF0) at each time point. Solid symbols and vertical lines represent the arithmetic mean and 90% confidence interval, respectively, of the model‐adjusted ΔΔQTcF within each plasma milvexian concentration decile.

The final model described the ΔΔQTcF well (Figure 4 and Figure S7). Estimated parameters (with 95% CI) of the model are shown in Table 4. The slope point estimate was positive but not significantly different from 0. The model predicted mean (90% CI) ΔΔQTcF at the geometric mean of plasma milvexian C_max_ on Day 4 following q12h administration of 100 mg as a capsule and 200 mg solution was 0.26 ms (−2.02, 2.53) and 1.73 ms (−1.20, 4.66), respectively, which were well below the threshold of 10 ms. Thus, concentration‐QTc modeling did not detect a significant relationship between ΔΔQTcF and plasma milvexian concentrations.

**Table 4:** Final Parameter Estimates From the Concentration‐QTc Analysis (Linear Mixed‐Effects Model) to Evaluate the Relationship Between ΔΔQTcF and Plasma Milvexian Concentration

Parameter	Unit	Estimate	SE	Lower 95% CI	Upper 95% CI	*P* Value
β_0_	ms	−0.495	1.35	−3.15	2.17	0.716
β_1_	ms/(µg/mL)	0.502	0.286	−0.061	1.07	0.0858
QTcF0	ms	−0.433	0.0464	−0.539	−0.324	<0.001
η_int_	ms	9.62	–	7.52	12	–
η_slp_	ms/(µg/mL)	1.84	–	1.41	2.33	–
*ε*	ms	7.07	–	6.88	7.27	–

β_0_, intercept; β_1_, slope of the linear concentration–effect relationship; *ε*, residual variability; η_int_, inter‐individual variability on the intercept; η_slp_, inter‐individual variability on the slope; CI, confidence interval; QTcF0, centered baseline QTcF; SE, standard error.

Model parameters were reported with a 95% CI for significance in the model.

#### Safety and Tolerability

All 66 (100.0%) participants enrolled in the study were included in the safety analysis set. At least one treatment‐emergent adverse event (TEAE) was reported in 63 (95.5%) participants: in 36 (65.5%) participants following administration of 100 mg milvexian, 46 (83.6%) participants following administration of 200 mg milvexian, 31 (53.4%) participants following administration of moxifloxacin, and 38 (66.7%) participants following placebo (Table 5). The most frequently reported nonbleeding TEAEs considered related to milvexian (or matched placebo) by the investigator (≥10.0% of participants in any treatment group) following administration of 100 mg milvexian, 200 mg milvexian, moxifloxacin, and placebo were headache, diarrhea, nausea, and abdominal pain. No deaths or other serious adverse events were reported in this study. There was no ECG abnormality which was reported as a TEAE. No severe TEAEs were reported. In 53 participants (80.3%), only mild TEAEs were reported, and 10 (15.2%) participants reported at least one moderate TEAE.

**Table 5:** Safety Summary

Participants with ≥1, n (%)	Placebo (n = 57)	Milvexian 100 mg (n = 55)	Milvexian 200 mg (n = 55)	Moxifloxacin (n = 58)	All Participants (N = 66)
All TEAEs	38 (66.7)	36 (65.5)	46 (83.6)	31 (53.4)	63 (95.5)
Serious TEAE	0	0	0	0	0
TEAE leading to death^*^	0	0	0	0	0
TEAE leading to study discontinuation^†^	2 (3.5)	2 (3.6)	5 (9.1)	1 (1.7)	12 (18.2)
TEAE leading to treatment discontinuation^‡^	2 (3.5)	2 (3.6)	4 (7.3)	1 (1.7)	10 (15.2)
TEAE (excluding COVID‐19 and asymptomatic COVID‐19) leading to treatment discontinuation^‡^	1 (1.8)	1 (1.8)	0	0	3 (4.5)
Common TEAEs, **≥**10% in any group					
Diarrhea	3 (5.3)	7 (12.7)	29 (52.7)	3 (5.2)	36 (54.5)
Headache	15 (26.3)	11 (20.0)	15 (27.3)	8 (13.8)	34 (51.5)
Abdominal pain	3 (5.3)	3 (5.5)	9 (16.4)	1 (1.7)	16 (24.2)
Nausea	4 (7.0)	4 (7.3)	7 (12.7)	2 (3.4)	12 (18.2)
Non‐bleeding TEAEs	37 (64.9)	35 (63.6)	46 (83.6)	30 (51.7)	63 (95.5)
Serious TEAE	0	0	0	0	0
TEAE leading to death^*^	0	0	0	0	0
TEAE leading to study discontinuation^†^	2 (3.5)	2 (3.6)	5 (9.1)	1 (1.7)	12 (18.2)
TEAE leading to treatment discontinuation^‡^	2 (3.5)	2 (3.6)	4 (7.3)	1 (1.7)	10 (15.2)
TEAE (excluding COVID‐19 and asymptomatic COVID‐19) leading to treatment discontinuation^‡^	1 (1.8)	1 (1.8)	0	0	3 (4.5)
Bleeding TEAEs	6 (10.5)	4 (7.3)	10 (18.2)	6 (10.3)	23 (34.8)
Serious TEAE	0	0	0	0	0
TEAE leading to death^*^	0	0	0	0	0
TEAE leading to study discontinuation^†^	0	0	0	0	0
TEAE leading to treatment discontinuation^‡^	0	0	0	0	0
TEAE (excluding COVID‐19 and asymptomatic COVID‐19) leading to treatment discontinuation^‡^	0	0	0	0	0

TEAE, treatment‐emergent adverse event.

* AEs leading to death is based on AE outcome of fatal.

^†^ The reason for study discontinuation is the TEAE declared in the CRF study termination form.

^‡^ Action taken regarding the TEAE is ‘drug withdrawal’ declared in the CRF AE form.

One participant (female, 28 years of age) had an adverse event of moderate rash on Day 3 of treatment with 200 mg milvexian solution. The TEAE of moderate rash was reported as related to study drug and led to study discontinuation. Another participant (male, 50 years of age) had a TEAE of mild hepatic enzyme increase with alanine aminotransferase (ALT), alkaline phosphatase (ALP), and gamma‐glutamyl transferase (GGT) elevation following 100 mg milvexian capsule (ALT 1.5× and GGT 1.6× upper limit of normal) and 200 mg milvexian solution (ALT 1.8×, ALP 1.1×, and GGT 2.5× upper limit of normal). This TEAE was reported by the investigator as related to study drug; however, the adverse event resolved without additional measure taken toward the study intervention.

At least one bleeding TEAE was reported in 23 (34.8%) participants: in 4 (7.3%) participants following 100 mg milvexian, 10 (18.2%) participants following 200 mg milvexian, 6 (10.3%) participants following moxifloxacin, and 6 (10.5%) participants following placebo. No severe bleeding TEAEs were reported. For 21 (31.8%) participants only mild bleeding TEAEs were reported. There were two moderate bleeding TEAEs which were classified as clinically relevant non‐major bleeding according to the International Society on Thrombosis and Hemostasis (ISTH) classification as well as Type 2 according the Bleeding Academic Research Consortium (BARC) scale. Both bleeding adverse events were vessel puncture site hematoma reported in 2 (3.0%) participants following administration of moxifloxacin. All other bleeding events were classified as ISTH type “other” and BARC Type 1. The most frequently reported bleeding TEAE (≥5.0%) was hematochezia in three (5.5%) participants following milvexian 200 mg; considered minor for all three participants.

## Discussion

This TQT study demonstrated that 100 and 200 mg of milvexian administered q12h to healthy participants did not have a clinically relevant effect on cardiac repolarization. For both regimens, the upper bounds of the two‐sided 90% CIs were <10 ms for all baseline‐adjusted, time‐matched mean differences in QTcF intervals between milvexian and placebo (i.e., ΔQTcF), thereby fulfilling the criteria for a negative QT/QTc study per ICH E14.^22^, ^23^ This finding was confirmed by the lack of a relationship between ΔΔQTcF and plasma milvexian concentrations, which was evaluated using a pre‐specified linear mixed‐effect model. For both regimens, the upper limit of the model‐predicted mean (90% CI) ΔΔQTcF at the mean of milvexian C_max_ on Day 4 was <10 ms. Further, categorical analyses did not yield any suspicious findings for milvexian (or placebo) with regard to the QTc interval prolongation. A single dose of 400 mg moxifloxacin confirmed that the study had adequate sensitivity to detect increases in QTc. In addition, wave morphology changes were not observed after single‐ and repeated‐dose administration of milvexian. The results of the present study are consistent with the absence of clinically important findings from ECGs in healthy participants and participants with hepatic or renal impairment who received either single doses or q12h regimens of milvexian.^14^, ^15^, ^16^, ^17^, ^18^, ^24^, ^25^

The pharmacokinetics of milvexian at the regimen of 100 mg administered q12h as a capsule in the present study was similar to that observed previously in healthy participants.^33^ The mean C_max_ of milvexian achieved by the supratherapeutic regimen of 200 mg q12h as an oral solution was approximately 3× higher than the mean C_max_ achieved with 100 mg q12h administered as a capsule. The greater than dose‐proportional increase in milvexian exposure aligned with results from a previous study demonstrated the oral bioavailability of milvexian is greater when administered as a solution relative to a capsule.^15^

Milvexian dose regimens were selected for the TQT study with the intention to achieve clinically relevant and supratherapeutic plasma milvexian concentrations relative to the ongoing LIBREXIA Phase 3 program, which includes oral milvexian regimens of 25 or 100 mg twice daily (ClinicalTrials.gov identifiers: NCT05702034, NCT05754957, and NCT05757869). The highest individual plasma milvexian C_max_ attained in the present TQT study (6290 ng/mL) exceeds the reported 95th percentile of simulated milvexian concentrations (<5000 ng/mL) over a 12‐h dosing interval under steady‐state conditions in patients who had undergone a total knee replacement and were treated with the highest clinically relevant regimen of milvexian (i.e., 100 mg twice daily).^34^ An even greater margin is estimated between milvexian concentrations achieved in the QT study and those that can be achieved in patients treated with 25 mg of milvexian twice daily.

The results of the TQT study are supported by results of in vitro experiments, which evaluated the effects of milvexian at clinically relevant concentrations (e.g., the lowest concentration tested, 3 µM, is close to the highest concentrations achieved in patients in a milvexian Phase 2 study^34^) on cardiac potassium (hERG/IKr), sodium, and L‐type calcium channel currents using corresponding recombinant ion channels that were stably expressed in human embryonic kidney cells as test systems. The inclusion of the in vitro study was important given that it could have provided information regarding the mechanism of action if QT prolongation had been observed. In the current study, milvexian inhibited cardiac hERG/IKr potassium, sodium, and L‐type calcium channel currents with only weak‐to‐moderate potency at test concentrations, which substantially exceeded the highest mean unbound maximum concentrations in plasma of participants in the TQT study and patients who have undergone a total knee replacement. The unbound concentration of milvexian may be estimated based on the total concentrations in plasma mentioned above and a free fraction in plasma of 0.0775.^17^

Administration of milvexian 100 or 200 mg q12h (doses equal to or greater than those used in the LIBREXIA Phase 3 program) for 4 days was generally safe and well tolerated by participants in the TQT study. Notably, no cardiovascular or ECG‐related adverse events were observed. The higher proportion of participants with adverse events of diarrhea and abdominal pain following the 200 mg of milvexian compared with the other treatments can be attributed to the presence of PEG 400 in the milvexian solution. In an earlier Phase 1 study (NCT022600), the reported frequency and intensity of gastrointestinal adverse events increased with increasing doses of PEG 400.^35^

The current TQT study was completed prior to the start of the ongoing pivotal Phase 3 studies in the LIBREXIA program. The results demonstrated that the pharmacological effect of milvexian on cardiac repolarization was below the threshold that would raise clinical or regulatory concern. Furthermore, the results indicating a “negative thorough QT/QTc study” support a standard approach of collecting on‐therapy ECGs in accordance with the current practices in target patient populations. Consequently, the study contributed to the body of evidence supporting the design of the milvexian Phase 3 studies.

## Author Contributions

All authors made substantial contributions to the study design, data acquisition, analysis, or interpretation of the data, drafted or revised the manuscript for intellectual content, provided final approval of the version to be published, and agree to be accountable for all aspects of the work.

## Conflicts of Interest

Peter Zannikos, JongHanne Park, Anna Dari, Samiha Takhtoukh, Alexei N. Plotnikov, Amitava  Mitra, Juan Jose Perez Ruixo, and Navin Goyal were employed by Johnson & Johnson at the time the study was conducted and, as such, may have been eligible for stock and stock options. Antoinette Ajavon‐Hartmann, Paul Levesque, and Samira Merali were employed by Bristol Myers Squibb at the time the study was conducted and, as such, may have been eligible for stock and stock options.

## Funding

This study was sponsored by Bristol Myers Squibb and Johnson & Johnson.

## Consent to Participate

Written informed consent was obtained from each participant before enrollment in the study after being advised of the potential risks and benefits of the study, as well as the investigational nature of the study.

## Supporting information



Supporting Information

## Data Availability

The data sharing policy of Johnson & Johnson is available at https://innovativemedicine.jnj.com/our‐innovation/clinical‐trials/transparency. Although these data are not currently publicly available for sharing, requests for sharing can be sent to the corresponding author and will be evaluated on an individual basis.

## References

[1] Lutsey PL , Zakai NA . Epidemiology and prevention of venous thromboembolism. Nat Rev Cardiol. 2023;20:248‐262. 36258120 10.1038/s41569-022-00787-6 PMC9579604

[2] Wendelboe A , Weitz JI . Global health burden of venous thromboembolism. Arterioscler Thromb Vasc Biol. 2024;44:1007‐1011. 38657032 10.1161/ATVBAHA.124.320151

[3] Wendelboe AM , Raskob GE . Global burden of thrombosis: epidemiologic aspects. Circ Res. 2016;118:1340‐1347. 27126645 10.1161/CIRCRESAHA.115.306841

[4] Chiarito M , Cao D , Cannata F , et al. Direct oral anticoagulants in addition to antiplatelet therapy for secondary prevention after acute coronary syndromes: a systematic review and meta‐analysis. JAMA Cardiol. 2018;3:234–241. 29417147 10.1001/jamacardio.2017.5306 PMC5885890

[5] Dentali F , Douketis JD , Gianni M , Lim W , Crowther MA . Meta‐analysis: anticoagulant prophylaxis to prevent symptomatic venous thromboembolism in hospitalized medical patients. Ann Intern Med. 2007;146:278‐288. 17310052 10.7326/0003-4819-146-4-200702200-00007

[6] Lip G , Freedman B , De Caterina R , Potpara TS . Stroke prevention in atrial fibrillation: past, present and future. Comparing the guidelines and practical decision‐making. Thromb Haemost. 2017;117:1230‐1239. 28597905 10.1160/TH16-11-0876

[7] Lloyd NS , Douketis JD , Moinuddin I , Lim W , Crowther MA . Anticoagulant prophylaxis to prevent asymptomatic deep vein thrombosis in hospitalized medical patients: a systematic review and meta‐analysis. J Thromb Haemost. 2008;6:405‐414. 18031292 10.1111/j.1538-7836.2007.02847.x

[8] Malik AH , Yandrapalli S , Aronow WS , Panza JA , Cooper HA . Meta‐analysis of direct‐acting oral anticoagulants compared with warfarin in patients >75 years of age. Am J Cardiol. 2019;123:2051‐2057. 30982541 10.1016/j.amjcard.2019.02.060

[9] Barra ME , Fanikos J , Connors JM , Sylvester KW , Piazza G , Goldhaber SZ . Evaluation of dose‐reduced direct oral anticoagulant therapy. Am J Med. 2016;129:1198‐1204. 27341955 10.1016/j.amjmed.2016.05.041

[10] Sterne JAC , Bodalia PN , Bryden PA , et al. Oral anticoagulants for primary prevention, treatment and secondary prevention of venous thromboembolic disease, and for prevention of stroke in atrial fibrillation: Systematic review, network meta‐analysis and cost‐effectiveness analysis. Health Technol Assess. 2017;21:1‐386. 10.3310/hta21090 PMC5366855 28279251

[11] Wang X , Li Q , Du F , Shukla N , Nawrocki AR , Chintala M . Antithrombotic effects of the novel small‐molecule factor XIa inhibitor milvexian in a rabbit arteriovenous shunt model of venous thrombosis. TH Open. 2023;7:e97‐e104. 37101592 10.1055/a-2061-3311 PMC10125780

[12] Wong PC , Crain EJ , Bozarth JM , et al. Milvexian, an orally bioavailable, small‐molecule, reversible, direct inhibitor of factor XIa: in vitro studies and in vivo evaluation in experimental thrombosis in rabbits. J Thromb Haemost. 2022;20:399‐408. 34752670 10.1111/jth.15588 PMC9299130

[13] Dilger AK , Pabbisetty KB , Corte JR , et al. Discovery of milvexian, a high‐affinity, orally bioavailable inhibitor of factor XIa in clinical studies for antithrombotic therapy. J Med Chem. 2022;65:1770‐1785. 34494428 10.1021/acs.jmedchem.1c00613

[14] Perera V , Wang Z , Luettgen J , et al. First‐in‐human study of milvexian, an oral, direct, small molecule factor XIa inhibitor. Clin Transl Sci. 2022;15:330‐342. 34558200 10.1111/cts.13148 PMC8841437

[15] Jarugula P , Soleman S , Back H , et al. Absolute oral bioavailability of milvexian spray‐dried dispersion formulation under fasted and fed conditions in healthy adult participants: an intravenous microtracer approach. Clin Transl Sci. 2024;17:e70058. 39450784 10.1111/cts.70058 PMC11503494

[16] Perera V , Wang Z , Lubin S , et al. Effects of itraconazole and diltiazem on the pharmacokinetics and pharmacodynamics of milvexian, a factor XIa inhibitor. Cardiol Ther. 2022;11:407‐419. 35641780 10.1007/s40119-022-00266-6 PMC9381674

[17] Perera V , Abelian G , Li D , et al. Single‐dose pharmacokinetics of milvexian in participants with mild or moderate hepatic impairment compared with healthy participants. Clin Pharmacokinet. 2022;61:857‐867 35262846 10.1007/s40262-022-01110-9 PMC9249726

[18] Perera V , Abelian G , Li D , et al. Single‑dose pharmacokinetics of milvexian in participants with normal renal function and participants with moderate or severe renal impairment. Clin Pharmacokinet. 2022;61:1405‐1416. 35906349 10.1007/s40262-022-01150-1 PMC9553801

[19] Weitz JI , Strony J , Ageno W , et al. Milvexian for the prevention of venous thromboembolism. N Engl J Med. 2021;385:2161‐2172. 34780683 10.1056/NEJMoa2113194 PMC9540352

[20] Sharma M , Molina CA , Toyoda K , et al. Safety and efficacy of factor XIa inhibition with milvexian for secondary stroke prevention (AXIOMATIC‐SSP): a phase 2, international, randomised, double‐blind, placebo‐controlled, dose‐finding trial. Lancet Neurol. 2024;23:46‐59. 38101902 10.1016/S1474-4422(23)00403-9 PMC10822143

[21] Crumb W , Cavero I . QT interval prolongation by non‐cardiovascular drugs: issues and solutions for novel drug development. Pharm Sci Technol Today. 1999;2:270‐280. 10407390 10.1016/s1461-5347(99)00172-8

[22] ICH Topic E14 The clinical evaluation of QT/QTc interval prolongation and proarrhythmic potential for non‐antiarrhythmic drugs. European Medicines Agency; 2005. Accessed October 6, 2025. https://www.ema.europa.eu/en/documents/scientific‐guideline/ich‐e‐14‐clinical‐evaluation‐qt/qts‐interval‐prolongation‐proarrhythmic‐potential‐non‐antiarrhythmic‐drugs‐step‐5_en.pdf

[23] E14 clinical evaluation of QT/QTc interval prolongation and proarrhythmic potential for non‐antiarrhythmic drugs—questions and answers (R3) . Guidance for Industry. US Department of Health and Human Services; US Food and Drug Administration; Center for Drug Evaluation and Research; Center for Biologics Evaluation and Research, 2017. Accessed June 26, 2025. https://collections.nlm.nih.gov/catalog/nlm:nlmuid‐101712951‐pdf. Assessed 06 October 2025.

[24] Perera V , Wang Z , Lubin S , et al. Effects of rifampin on the pharmacokinetics and pharmacodynamics of milvexian, a potent, selective, oral small molecule factor XIa inhibitor. Sci Rep. 2022;12:22239. 36564395 10.1038/s41598-022-25936-2 PMC9789074

[25] Perera V , Wang, Z , Lubin S , et al. Safety, pharmacokinetics, and pharmacodynamics of milvexian in healthy Japanese participants. Sci Rep. 2022;12:5165. 35338177 10.1038/s41598-022-08768-y PMC8956633

[26] Shih HT . Anatomy of the action potential in the heart. Tex Heart Inst J. 1994;21:30‐41. 7514060 PMC325129

[27] Grant AO . Cardiac ion channels. Circ Arrhythm Electrophysiol. 2009;2:185‐194. 19808464 10.1161/CIRCEP.108.789081

[28] Salvi V , Karnad DR , Panicker GK , Kothari S . Update on the evaluation of a new drug for effects on cardiac repolarization in humans: issues in early drug development. Br J Pharmacol. 2010;159:34‐48. 19775279 10.1111/j.1476-5381.2009.00427.x PMC2823350

[29] Bloomfield DM , Kost JT , Ghosh K , et al. The effect of moxifloxacin on QTc and implications for the design of thorough QT studies. Clin Pharmacol Ther. 2008;84:475‐480. 19238652 10.1038/clpt.2008.33

[30] AVELOX Summary of Product Characteristics . Brussels: Bayer s.a.‐n.v. Accessed 06 October 2025. https://www.ema.europa.eu/en/documents/referral/avelox‐article‐6‐12‐referral‐annex‐i‐ii‐iii_en.pdf

[31] Malik M , Garnett C , Hnatkova K , et al. Implications of individual QT/RR profiles—part 1: inaccuracies and problems of population specific QT/heart rate corrections. Drug Saf. 2018;42:401‐414. 10.1007/s40264-018-0736-1 PMC6426828 30255349

[32] Garnett C , Bonate PL , Dang Q , et al. Scientific white paper on concentration‐QTc modeling. J Pharmacokinet Pharmacodyn. 2018;45:383‐397. 29209907 10.1007/s10928-017-9558-5

[33] Zannikos P , Merali S , Mitra A , et al. Pharmacokinetics and pharmacodynamics of milvexian after administration every 12 hours in healthy subjects. Paper presented at: American College of Clinical Pharmacology (ACCP) annual meeting, September 8–10, 2024; Bethesda, MD.

[34] Zhou W , Bozenhardt E , Alexander GE , et al. Quantitative model‐informed dose selection for a milvexian phase III study in patients with atrial fibrillation. Clin Pharmacol Ther. 2025;119(1):139‐149. 10.1002/cpt.70037 40844380 PMC12746510

[35] Clinical Study Synopsis for Public Disclosure. Safety of total daily doses of polyethylene glycol (PEG) 400 administered orally to healthy male human subjects. Boehringer Ingelheim Pharma KG BI Trial No. 352.2030. Assessed 06 October 2025. https://www.mystudywindow.com/trial/completed/253791/0352‐2030

