# Supplementary material for: A Thorough QT Study to Assess the Effects of Milvexian on Cardiac Repolarization in Healthy Participants
Source: Clin Pharmacol Drug Dev. 2026 Jan 19;15(1):e70015. doi: 10.1002/cpdd.70015 (PMC12813654; doi:10.1002/cpdd.70015)
Supplement: Supplementary file 1 — Supporting Information [file CPDD-15-0-s001.docx]

**Supplemental Material**

**Supplemental methodology: Description of assays used to assess the effects of milvexian on potassium, sodium, and L-type calcium currents**

**Potassium currents**: The ion channel responsible for the rapid component of the cardiac delayed rectifier potassium current (IKr) is encoded by the potassium channel gene, human ether–a-go-go-related gene (hERG).^1^ Human embryonic kidney (HEK293) cells stably transfected with hERG cDNA were used in the hERG assay. The hERG bath solution, which replaced the cell culture media during experiments, contained (in mM): 140 NaCl, 4 KCl, 1.8 CaCl_2_, 1 MgCl_2_, 10 glucose, 10 HEPES (pH 7.4, NaOH). Borosilicate glass pipettes had tip resistances of 2 to 4 MΩ when filled with an internal solution containing (in mM): 130 KCl, 1 MgCl_2_, 1 CaCl_2_, 5 ATP-K_2_, 10 EGTA, 10 HEPES (pH 7.2, KOH).

While perfusing with control bath solution, repetitive test pulses (0.05 Hz) were applied from a holding potential of –80 mV to +20 mV for 2 seconds. Tail currents were elicited following the test pulses by stepping the voltage to –65 mV for 3 seconds. After recording the steady-state current for 2 to 5 minutes in the absence of test article (control), the bath solution was switched to one containing the lowest concentration of milvexian to be used. The peak tail current was monitored until a new steady state in the presence of milvexian was achieved. This was followed by the application of the next higher concentration of milvexian to be tested and was repeated until all concentrations of milvexian had been evaluated. The effects of milvexian on hERG

channel were calculated by measuring inhibition of peak tail currents. Percent inhibition of tail currents was plotted as a function of milvexian concentration to quantify hERG channel inhibition.

**Sodium currents**: Sodium currents were studied using HEK293 cells stably expressing the cloned human sodium channel gene SCN5A. The sodium current bath solution contained (in mM): 140 NaCl, 4 KCl, 1.8 CaCl_2_, 1 MgCl_2_, 10 glucose, 10 HEPES (pH 7.4, NaOH). The patch pipette filling solution used in sodium experiments contained (in mM): 130 KCl, 1 MgCl_2_, 1 CaCl_2_, 5 ATP-K_2_, 10 EGTA, 10 HEPES (pH 7.2, KOH). For determining steady state inhibition, sodium currents were elicited every 5 seconds (0.2 Hz) using the following voltage protocol. Cells were held at a potential of –90 mV and stepped to –20 mV for 45 msec. The peak sodium current in response to the depolarizing step to –20 mV was monitored in the control buffer and after application of test article until a new steady state in the presence of the test article was achieved. To assess the rate-dependent inhibition of the sodium currents, trains of voltage steps at frequencies of 1 and 4 Hz (30 sweeps each) were applied to the cell prior to application of test article (control) and after steady state inhibition by test article, as determined at 0.2 Hz frequency. The voltage waveform used in the rate dependence experiments was the same as the waveform used for evaluating steady state inhibition at 0.2 H stimulation frequency. Rate dependent inhibition was calculated by comparing the 30^th^ voltage sweep in presence of test article to the 30^th^ voltage sweep under control conditions at each frequency tested.

**L-type calcium currents**: L-type calcium currents were studied using HEK293 cells stably expressing the cloned human cardiac L-type calcium channel (Cav1.2) α1C and β2 subunits.

The L-type calcium current bath solution contained (in mM): 103 NaCl, 30 BaCl_2_, 4 CsCl, 1 MgCl_2_, 10 glucose, 10 HEPES (pH 7.35, NaOH). Borosilicate glass pipettes had tip resistances of 2 to 4 MΩ when filled with an internal solution containing (in mM): 20 CsCl, 20 TEA chloride, 82 glutamate, 3 ATP-Mg, 0.5 NaH2PO4, 3 Na2-creatine PO_4_, 11 EGTA, 10 HEPES (pH 7.25, CsOH). The bath and the pipette solution in the L-type calcium channel assay minimized current rundown over time. L-type calcium currents were elicited by 200 msec step depolarizations applied from a holding potential of –50 mV to a test potential of +30 mV. The voltage steps were applied in 10-second intervals (0.1 Hz) and the peak inward current was recorded. After recording the steady state current for 2 to 5 minutes in the absence of test article, the bath solution was switched to one containing the lowest concentration of the test article to be tested. The peak inward current was monitored until a new steady state in the presence of test article was achieved. The percentage of inhibition of peak inward current at +30 mV was used to calculate the percentage inhibition of L-type calcium channel.

Milvexian was tested in 3 cells at each concentration in all ion channel assays. Currents were sampled at rates ≥2× the low pass filter rate. The flow rate was kept constant throughout the experiments. All currents were recorded at approximately 25^o^C. IC_50_ values were calculated in XLfit using the sigmoidal concentration-response equation:

Y=A+((B-A)/(1+((C/X)^D)))

where A and B are the minimum and maximum percent inhibition, C is the IC_50,_ and D is the slope factor.

1. Trudeau MC, Warmke JW, Ganetzky B, Robertson GA. HERG, a human inward rectifier in the voltage-gated potassium channel family. *Science*. 1995;269:92-95.

**Supplemental Table 1. Inclusion and exclusion criteria**

| **Inclusion criteria**  Each potential participant must have satisfied all of the criteria below to be enrolled in the study |
| --- |
| **Age** |
| - 18 to 55 years of age, inclusive, at the time of screening |
| **Type of participant and disease characteristics** |
| - Healthy on the basis of physical examination, medical history, and vital signs performed at screening and on Day –1 of Period 1. If there were abnormalities, the investigator could have decided that the abnormalities or deviations from normal were not clinically significant, in which case the participant may have been included |
| - Healthy on the basis of clinical laboratory tests performed at screening and on Day –1 of Period 1. If the results of the clinical laboratory tests were outside the normal reference ranges, the participant may have been included if the investigator judged the abnormalities or deviations from normal to be not clinically significant |
| **Weight** |
| - BMI (weight [kg]/height^2^ [m^2^]) between 18.0 and 30.0 kg/m^2^ (inclusive), and body weight not less than 50 kg at screening and Day –1 of the first treatment period |
| **Sex and contraceptive/barrier requirements** |
| - Man or woman (according to their reproductive organs and functions assigned by chromosomal complement) |
| - All women were required to have a negative highly sensitive serum (β-hCG) at screening and a urine pregnancy test on Day –1 of each treatment period |
| - Before randomization, a woman must have been: |
| - Not of childbearing potential |
| - Of childbearing potential and practicing a highly effective method of contraception (failure rate of <1% per year when used consistently and correctly) for ≥3 months prior to the study entry and agreed to remain on a highly effective method throughout the study and for ≥34 days after the last dose of study intervention |
| - A woman must have agreed not to donate eggs (ova, oocytes) for the purposes of assisted reproduction during the study and for a period of ≥34 days after the last study intervention administration |
| - During the study, a male who was sexually active with a female of childbearing potential or with a female who is pregnant must have agreed to use a barrier method of contraception (eg, condom with spermicidal foam/gel/film/cream/suppository) for a minimum of 94 days after receiving the last dose of study intervention |
| - A male participant must have agreed not to donate sperm for the purpose of reproduction during the study and for ≥94 days after receiving the last dose of study intervention |
| **Informed consent** |
| - Must have signed an ICF indicating that the participant understands the purpose of, and procedures required for, the study and was willing to participate in the study |
| **Other inclusions** |
| - Blood pressure (after the participant is supine for 5 minutes) between 90 and 140 mm Hg systolic, inclusive, and no higher than 90 mm Hg diastolic at screening and on Day –1 of Period 1 - Willing and able to adhere to the lifestyle restrictions and study procedures specified in the protocol |
| **Exclusion criteria**  Any potential participant who met any of the criteria listed below was excluded from participating in the study |
| **Medical conditions** |
| - History of any known illness at screening that, in the opinion of the investigator, might have confounded the results of the study or pose an additional risk in administering study intervention to the participant or that could prevent, limit or confound the protocol-specified assessments. This could have included but was not limited to any known bleeding or clotting disorder; a history of arterial or venous thrombosis, liver or renal dysfunction, significant cardiac, vascular, pulmonary, gastrointestinal, endocrine, neurologic, hematologic, rheumatologic, psychiatric, neoplastic abnormalities, metabolic disturbances, or poor venous access; history of risk factors for TdP; family history of short QT syndrome, long QT syndrome, sudden unexpected death at age ≤40 years, drowning or sudden infant death syndrome in a first-degree relative (ie, biologic parent, sibling, or child); or a history of unexplained syncopal episodes |
| - Any skin condition likely to have interfered with electrocardiographic electrode placement or adhesion at screening and Day –1 of Period 1 |
| - Breast implant or a history of thoracic surgery likely to have caused abnormality of the electrical conduction through thoracic tissues at screening |
| - Clinically relevant bleeding history, including history of excessive menstrual bleeding in women, as determined by the investigator or appropriate designee, at screening |
| - Clinically significant abnormal values for hematology, coagulation, hypokalemia, hypocalcemia, clinical chemistry or urinalysis at screening or on Day –1 of Period 1 as determined by the investigator or appropriate designee. Any of the laboratory results listed below that were outside of the normal ranges specified at screening or Day –1 of Period 1 must have been confirmed by repeat: - Hemoglobin or hematocrit <lower limit of normal - Platelet count <lower limit of normal - aPTT or PT >1.2× upper limit of normal |
| - Any of the following on a 12-lead ECG and the assessment of QT interval, confirmed by repeat at screening and Day –1 of Period 1: - Heart rate >100 bpm or <45 bpm - PR >200 milliseconds - QRS >110 milliseconds - QTcF >450 milliseconds (Fridericia correction) - Clinically significant abnormal ECG intervals - ≥2 premature complexes over 30 seconds of the triplicate 12-lead safety ECGs |
| - History of any clinically significant drug or food allergies known allergy to the study interventions or any of the excipients of the formulation at screening |
| - History of allergy to or unwillingness to consume any component of the standard breakfast menu to be provided in this study |
| **Prior/concomitant therapy** |
| - Use of any prescription or nonprescription medication (including vitamins and herbal supplements), except for paracetamol/acetaminophen, hormonal contraceptives, and HRT (for female participants approved by the investigator) within 14 days before the first dose of study intervention in Period 1 until completion of the study |
| - Use of any systemic strong cytochrome P450 3A4 inducers or inhibitors within 4 weeks before the first dose of study intervention in Period 1 - Use of any systemic strong inducers or inhibitors of P-glycoprotein within 4 weeks before the first dose of study intervention in Period 1 |
| - Use of any agent that is known to increase the potential risk of bleeding within 2 weeks prior to the first dose of study intervention in Period 1 |
| **Prior/concurrent clinical study experience** |
| - Received an investigational intervention (including investigational vaccines) or used an invasive investigational medical device within 60 days before the first dose of study intervention in Period 1 or received an investigational biologic product within 3 months or 6 half-lives, whichever is longer, before the first dose of study intervention in Period 1 or was currently enrolled in an investigational study |
| - Pregnant, breast-feeding, or was planning to become pregnant before the first dose of the study intervention in Period 1, during the study, or within 34 days after the last dose of study intervention |
| - Any condition for which, in the opinion of the investigator, participation would not be in the best interest of the participant (eg, compromise the well-being) or that could prevent, limit, or confound the protocol-specified assessments |
| **Diagnostic assessments** |
| - Participants with current hepatitis B infection (confirmed by HBsAg), or hepatitis C infection (confirmed by HCV antibody), or HIV-1 or HIV-2 infection at screening |
| - Preplanned surgery or procedures that would interfere with the conduct of the study |
| **Other exclusions** |
| - Employee of the investigator or study site, with direct involvement in the study or other studies under the direction of that investigator or study site, as well as family members of the employees or the investigator |
| - History of, or a reason to believe a participant had a history of, drug or alcohol abuse according to DSM-V criteria within 1 year before screening which in the investigator’s opinion would compromise participant’s safety and/or compliance with the study procedures - Positive test result(s) for alcohol and/or drugs of abuse (including barbiturates, opiates, opioids, cocaine, cannabinoids, amphetamines, lysergic acid diethylamide, and benzodiazepines) at screening and/or Day –1 of Period 1 - Did not tolerate venipuncture - Donated blood or blood products or had substantial loss of blood (more than 500 mL) within 3 months before the first administration of study intervention or intention to donate blood or blood products during the study |
| - Unable to swallow solid, oral dosage forms whole with the aid of water (participants were not permitted to chew, divide, dissolve, or crush the study intervention) |
| - Use of tobacco- or nicotine-containing products (including, but not limited to: cigarettes, pipes, cigars, chewing tobacco, nicotine patches, nicotine lozenges, or nicotine gum), or excessive caffeine (more than 500 mg/day caffeine as contained in 5 cups of tea or coffee, or 8 cans of cola) within 6 months prior to the first dose of study intervention in Period 1 |

aPTT, activated partial thromboplastin time; β-hCG; β-human chorionic gonadotropin; BMI, body mass index; bpm, beats per minute; DSM-V, Diagnostic and Statistical Manual of Mental Disorders (5th edition); ECG, electrocardiogram; HBsAg, hepatitis B surface antigen; HCV, hepatitis C virus; HIV-1, human immunodeficiency virus Type 1; HIV-2, human immunodeficiency virus Type 2; HRT, hormone replacement therapy; ICF, informed consent form; PT, prothrombin time; TdP, torsades de pointes.

**Supplemental Table 2. Participant demographics and baseline characteristics**

| **Characteristic** | **ADBC**  **(n = 16)** | **BACD**  **(n = 17)** | **CBDA**  **(n = 16)** | **DCAB**  **(n = 17)** | **All participants**  **(N = 66)** |
| --- | --- | --- | --- | --- | --- |
| Age, y | 35.7 (12.3) | 36.6 (11.3) | 33.9 (11.8) | 35.6 (9.2) | 35.5 (11.0) |
| Weight, kg | 78.0 (11.5) | 76.3 (11.5) | 70.0 (9.6) | 70.2 (10.4) | 73.6 (11.1) |
| Height, cm | 173.7 (7.8) | 172.8 (7.6) | 169.2 (9.7) | 167.1 (11.3) | 170.7 (9.4) |
| BMI, kg/m^2^ | 25.8 (3.1) | 25.5 (3.1) | 24.4 (2.6) | 25.1 (2.3) | 25.2 (2.8) |
| Sex, n (%) |  |  |  |  |  |
| Female | 9 (56.3) | 6 (35.3) | 9 (56.3) | 11 (64.7) | 35 (53.0) |
| Male | 7 (43.8) | 11 (64.7) | 7 (43.8) | 6 (35.3) | 31 (47.0) |
| Race, n (%) |  |  |  |  |  |
| American Indian or Alaska Native | 0 | 1 (5.9) | 0 | 1 (5.9) | 2 (3.0) |
| Asian | 0 | 1 (5.9) | 1 (6.3) | 0 | 2 (3.0) |
| Black or African American | 2 (12.5) | 1 (5.9) | 1 (6.3) | 1 (5.9) | 5 (7.6) |
| White | 14 (87.5) | 14 (82.4) | 14 (87.5) | 15 (88.2) | 57 (86.4) |
| Ethnicity, n (%) |  |  |  |  |  |
| Hispanic | 1 (6.3) | 1 (5.9) | 0 | 0 | 2 (3.0) |
| Not Hispanic or Latina | 15 (93.8) | 16 (94.1) | 16 (100) | 17 (100) | 64 (97.0) |

Values are arithmetic means (SD) unless otherwise noted.

BMI, body mass index; SD, standard deviation.

**Supplemental Figure 1. Overview of study design**


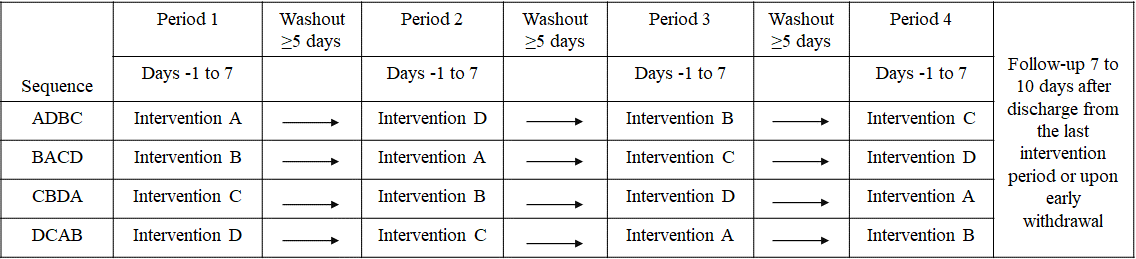


Intervention A: Milvexian 100 mg capsule and milvexian solution-matched placebo every 12 hours on Days 1 to 4 plus moxifloxacin-matched placebo on Day 4

Intervention B: Milvexian 200 mg solution and milvexian capsule-matched placebo every 12 hours on Days 1 to 4 plus moxifloxacin-matched placebo on Day 4

Intervention C: Milvexian capsule-matched placebo and milvexian solution-matched placebo every 12 hours on Days 1 to 4 plus moxifloxacin-matched placebo on Day 4

Intervention D: Milvexian capsule-matched placebo and milvexian solution-matched placebo every 12 hours on Days 1 to 4 plus moxifloxacin 400 mg on Day 4

**Supplemental Figure 2. Overview of study interventions**


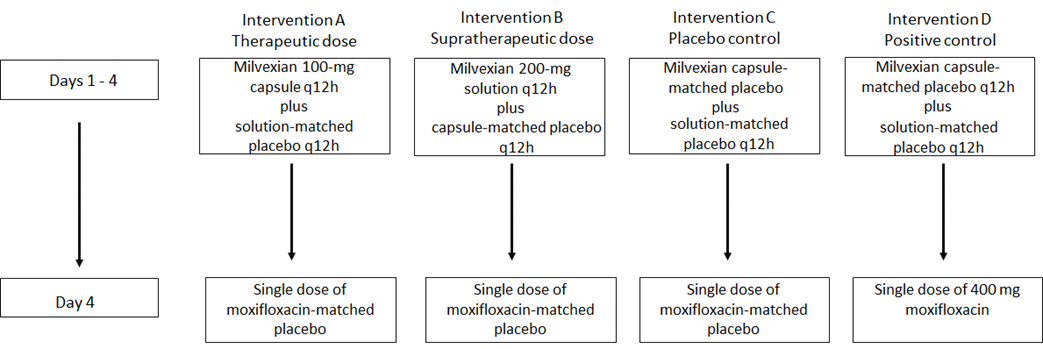


q12h, every 12 hours.

**Supplemental Figure 3. Arithmetic mean (unidirectional SD) plasma milvexian concentration-time profiles after every 12 hours oral administration for four days of 100 mg (capsule) and 200 mg (oral solution) of milvexian**

h, hour; SD, standard deviation.

**Supplemental Figure 4. Arithmetic mean (unidirectional 95% confidence interval) change from baseline over time in heart rate after administration of milvexian or placebo (A) on Day 1 or (B) Day 4.**

**A.**


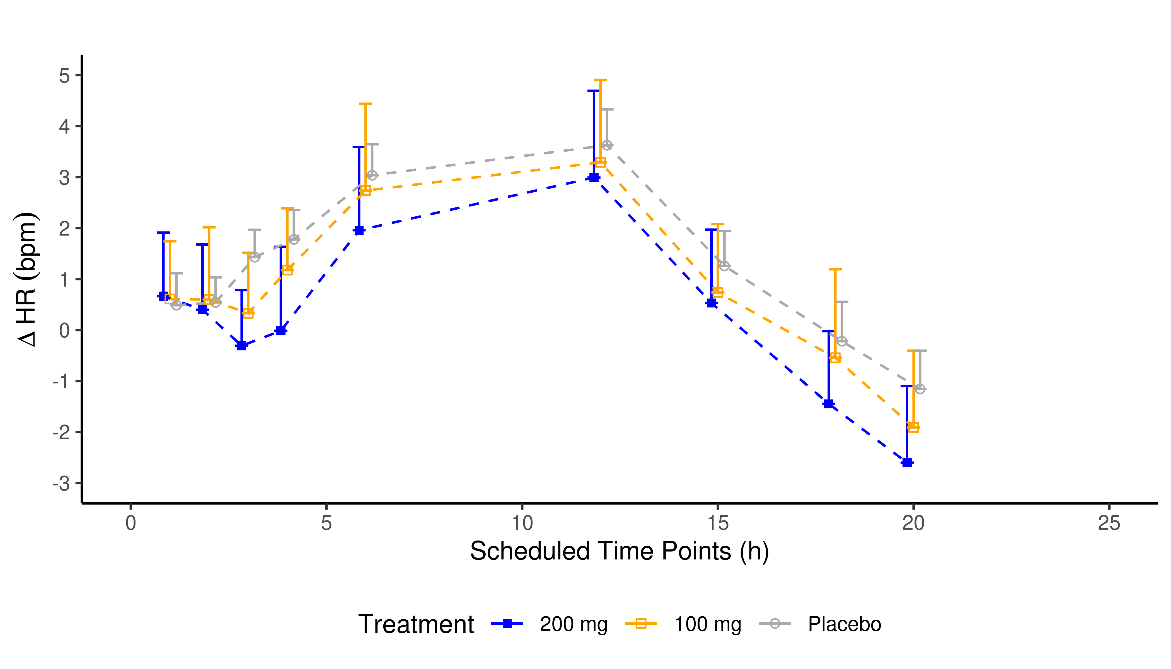


**B.**


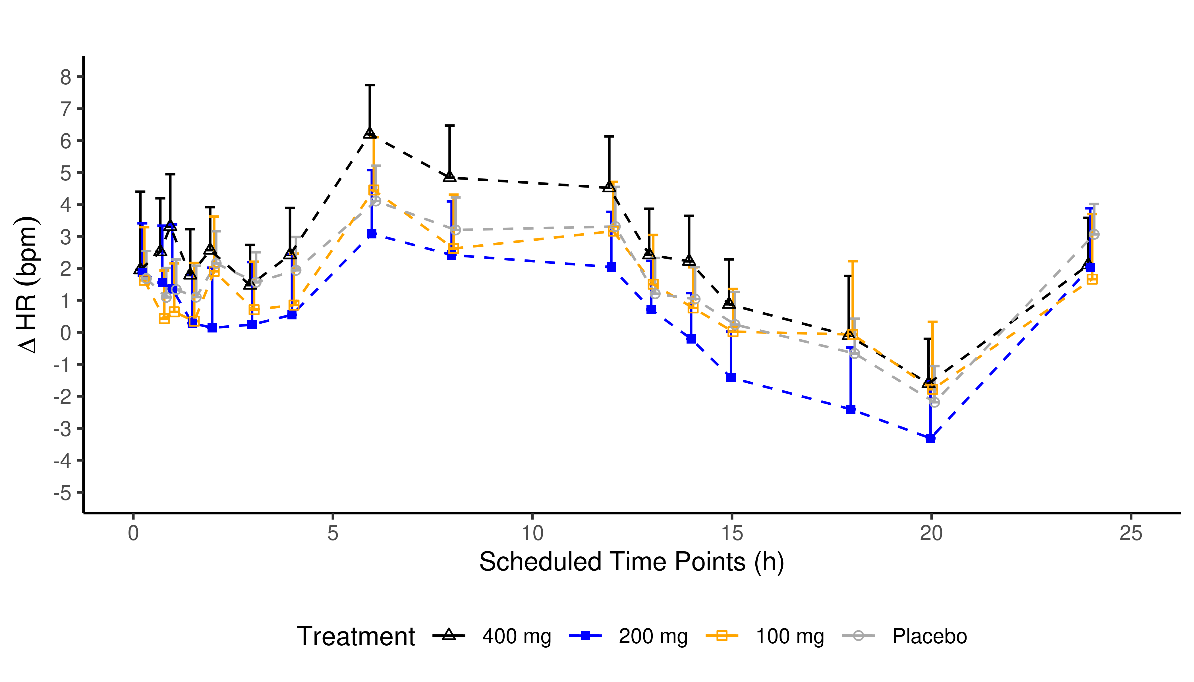


The 400 mg plot represents moxifloxacin. The 200 mg and 100 mg plots represent milvexian.

**Supplemental Figure 5. Graphical overlay of mean plasma milvexian concentrations and ΔΔQTcF as a function of time since first dose on (A) Day 1 and (B) Day 4, stratified by dose level.**

**A.**


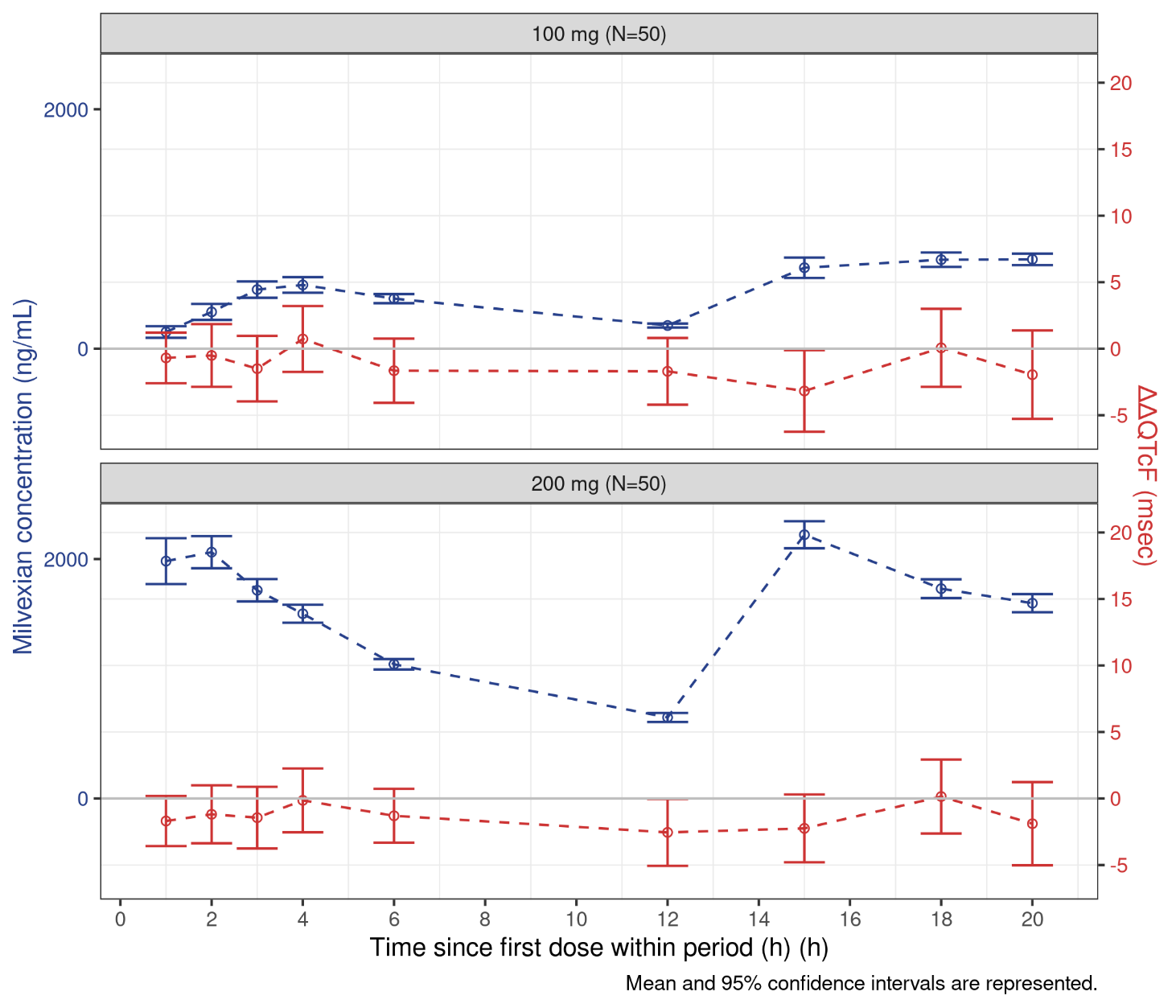


**B.**


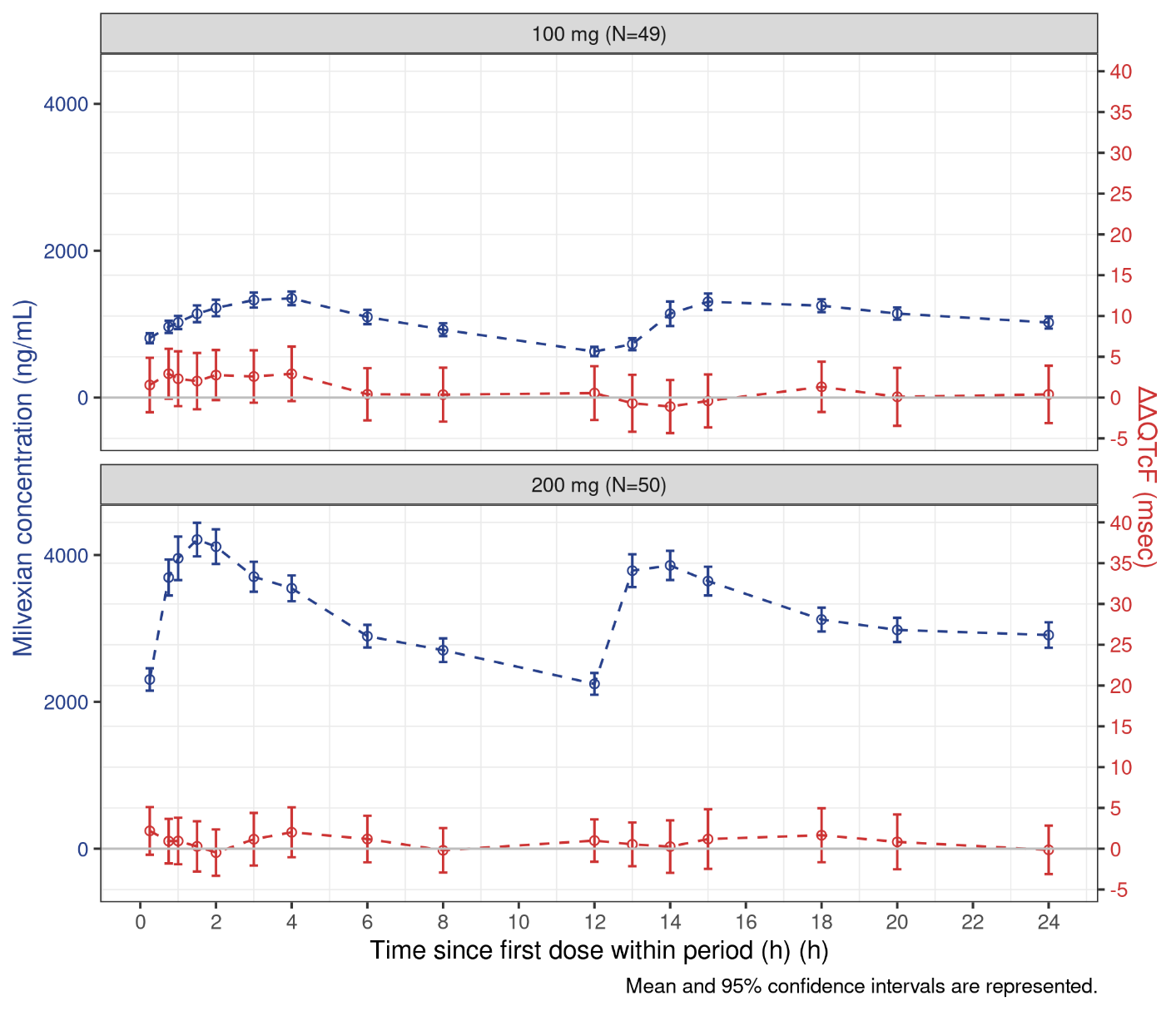


Time course of plasma milvexian concentration (blue, left y-axis) and observed ΔΔQTcF (red, right y-axis) by time point for each dose treatment. Open circles represent the corresponding arithmetic mean for each timepoint, and vertical lines represent 95% confidence interval.

**Supplemental Figure 6. Arithmetic mean (95% confidence interval) hysteresis plots representing the time ascending observed ΔΔQTcF versus plasma milvexian concentrations**


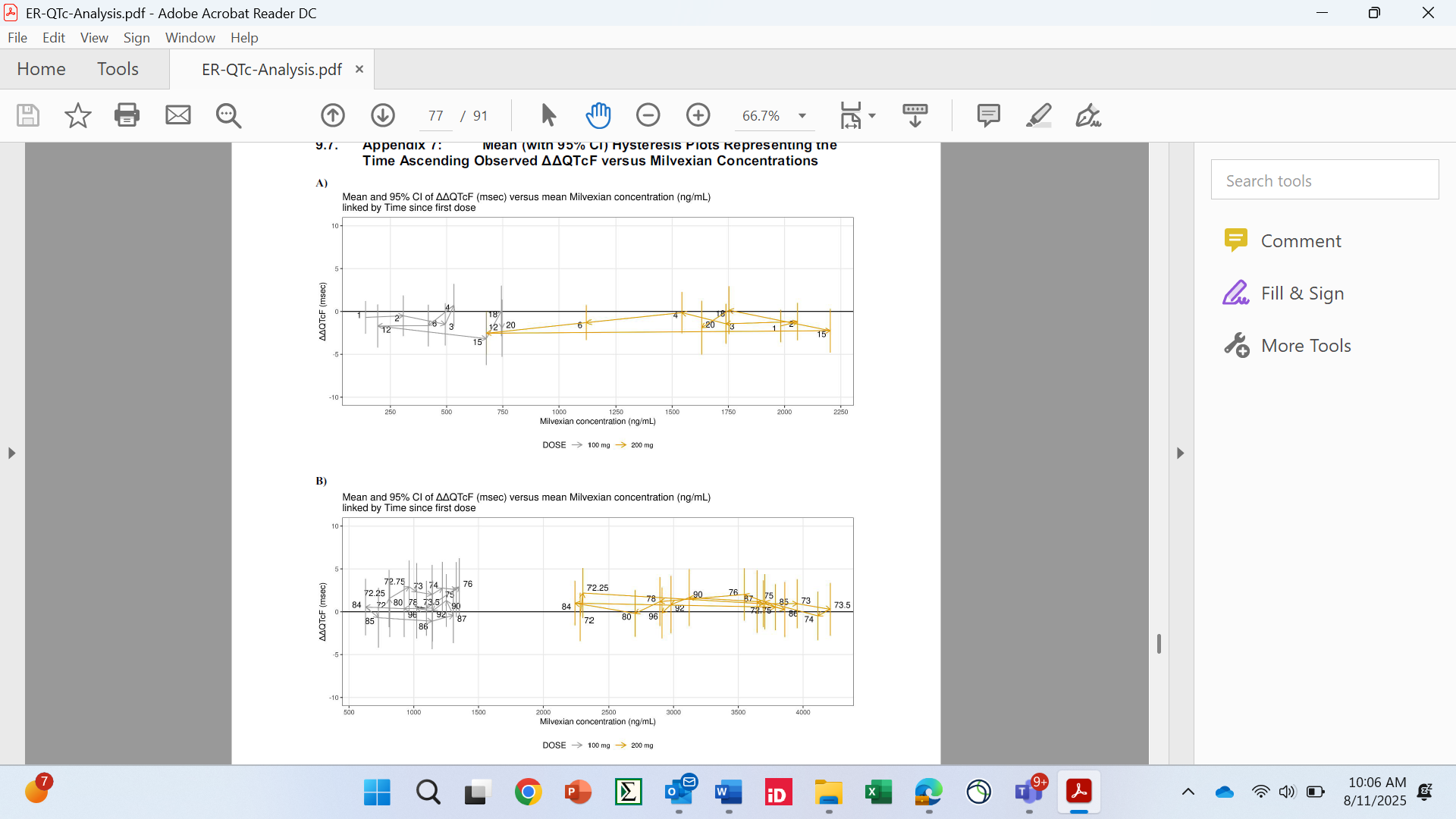


**Supplemental Figure 7. Goodness-of-fit plots of final exposure-response model with plasma milvexian concentration and ΔΔQTcF**

| **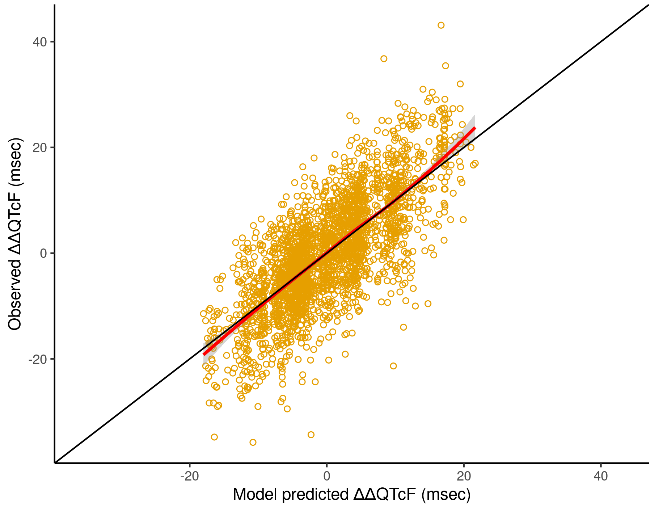** | **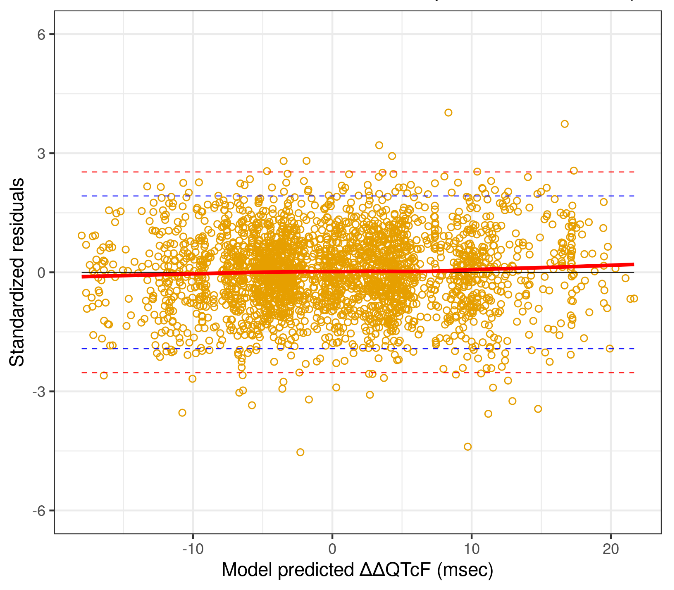** |
| --- | --- |
| **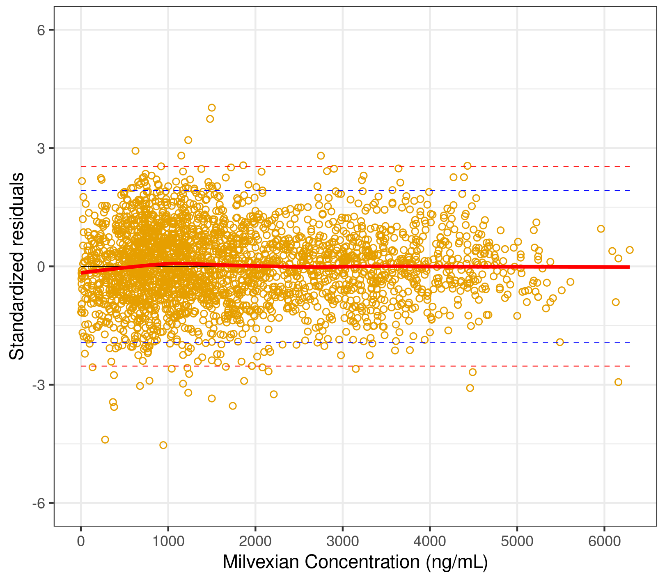** | **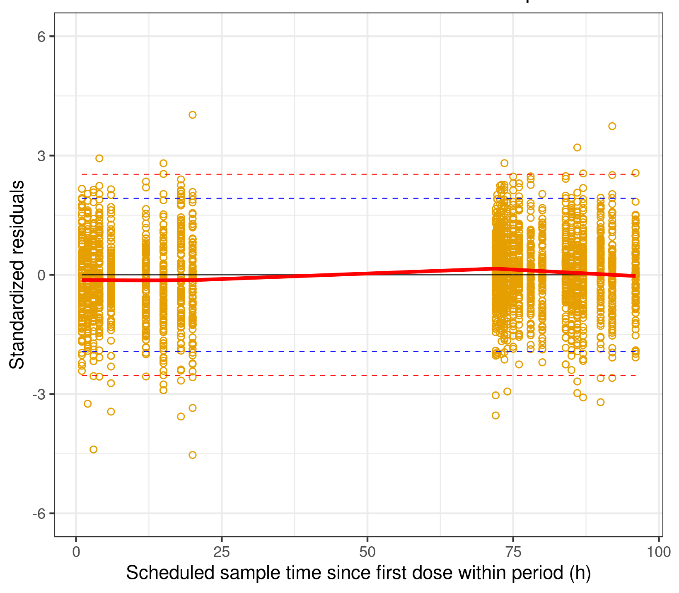** |
| **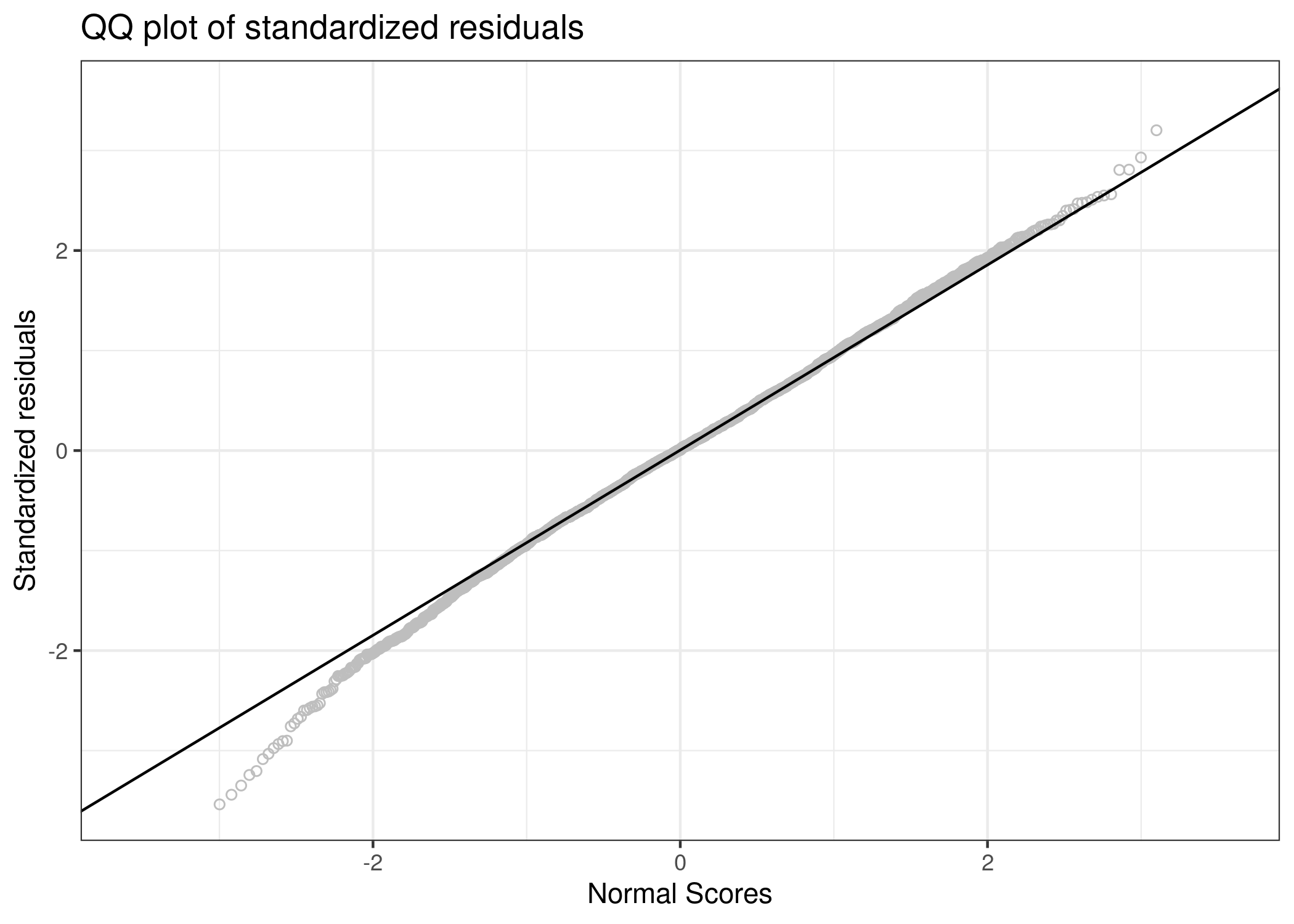** | |

Red dashed lines represent 99%CI. Blue dashed lines represent 95%CI. Red solid line represents the loess smooth line. Orange dots represent observations.
